# Genomic Data Reveals Cryptic Diversity in the Soda Lake Cichlid *Oreochromis amphimelas*

**DOI:** 10.1002/ece3.72054

**Published:** 2025-09-11

**Authors:** Miranda B. Sherlock, Emily Phelps, Kenji Yip Tong, Ewan H. Bodenham, Asilatu H. Shechonge, Antonia G. P. Ford, George F. Turner, Martin J. Genner, Julia J. Day

**Affiliations:** ^1^ Department of Genetics, Evolution and Environment University College London London UK; ^2^ Institute of Vertebrate Biology, Czech Academy of Sciences Brno Czech Republic; ^3^ Conservation and Ecology University of Exeter Penryn UK; ^4^ School of Biological Sciences University of East Anglia Norwich UK; ^5^ Department of Earth Sciences University College London London UK; ^6^ Tanzania Fisheries Research Institute (TAFIRI) Dar es Salaam Tanzania; ^7^ Department of Life Sciences, Whitelands College University of Roehampton London UK; ^8^ School of Environmental & Natural Sciences Bangor University Bangor Gwynedd UK; ^9^ School of Biological Sciences University of Bristol Bristol UK

**Keywords:** African freshwaters, cryptic species, morphological divergence, population genetics, tilapia

## Abstract

The cichlid fishes of East Africa are renowned for their rapid and species‐rich adaptive radiations. However, some specialist cichlid lineages in the region have not undergone extensive diversification; it is plausible that these lineages contain cryptic allopatric diversity. The tilapiine cichlid *Oreochromis amphimelas* (Hilgendorf, 1905) is a distinctive species specialised for high salinity, high alkalinity and high temperature soda lakes in the East African Rift Valley. Here, we investigated variation among *O. amphimelas* populations using a combination of reduced‐representation genome sequences, mitochondrial DNA sequences and morphological data. Genetic data revealed two highly divergent genomic lineages, with no evidence of ongoing gene flow. Specifically, the Lake Manyara population was strongly differentiated from the four other populations studied, which show relatively low levels of genetic differentiation among them. Genetic differentiation between Lake Manyara and the other populations is seen across the genome, as characterised by elevated windowed *F*_ST_. Despite the clear genomic divergence between the Lake Manyara and other soda lake populations, there were no apparent morphological differences between the two lineages, indicating they may be considered two cryptic species. It is possible that *O. amphimelas* lineages have diverged vicariantly following regional geomorphological change. Identification of two potential geographically separated cryptic species in the lineage has conservation implications, given that *O. amphimelas* is currently categorised as Endangered on the IUCN Red List of Threatened Species, due to threats from fisheries and environmental change.

## Graphical abstract

The cichlid fishes of East Africa are renowned for their rapid and species‐rich adaptive radiation; however, some lineages have not undergone extensive diversification, and it is plausible that these lineages contain cryptic allopatric diversity. Here, we investigated variation among populations of the soda lake tilapiine cichlid *O. amphimelas* using a combination of reduced‐representation genome sequences, mitochondrial DNA sequences and morphological data. Genetic data revealed two highly divergent genomic lineages, despite highly conserved morphology and it is likely that this split between lineages results from vicariance of the lakes caused by geographical features of the Tanzanian rift system.

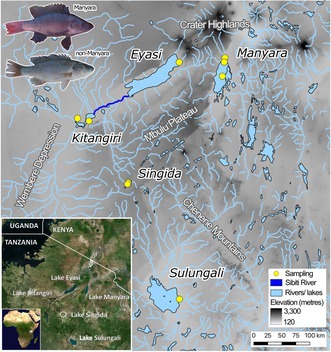

## 1 Introduction

Clarifying why diversity does or does not arise within a lineage is central to evolutionary biology (Alfaro et al. 2009; Marie Curie SPECIATION Network 2012). However, identifying the core processes that determine the rate of evolutionary diversification can be complex (Alfaro et al. 2009). East African lacustrine cichlids are a model system for understanding the processes driving the evolution of species diversity, due to their often high rates of diversification and elevated richness of sympatric species (Seehausen 2006; Seehausen 2015). The factors contributing to this diversity are complex and numerous (Sturmbauer et al. 2011; Turner 2007). These include macroecological factors that have influenced ecological opportunity, such as lake depth, energy availability, habitat complexity and lake area (Wagner et al. 2012; Malinsky et al. 2015; Seehausen 2006, 2015). They also include factors such as sexual selection (Kocher 2004), ecologically mediated behaviours (Sommer‐Trembo et al. 2024), interspecific hybridization (Irisarri et al. 2018; Svardal et al. 2020; Meier et al. 2023) and the presence of derived morphological innovations—including specialised pharyngeal jaws (Liem 1973) and sexually dimorphic anal fin phenotypes (Salzburger et al. 2005). Recent genomic studies have further demonstrated the significance of standing genetic variation and genomic flexibility in cichlid adaptive radiation, including gene duplication and high coding sequence evolution rates (Brawand et al. 2014; Seehausen 2015; Salzburger 2018). However, the reasons why some lineages exhibit high degrees of morphological and ecological disparity (Albertson 2008; Ford et al. 2016) whilst others have failed to radiate (Wagner et al. 2012) remain unclear in many cases. Indeed, the instances in which lacustrine cichlid lineages have failed to radiate can exceed the cases where extensive diversification has taken place (Seehausen 2006; Wagner et al. 2012).

The factors facilitating large‐scale adaptive radiation, such as ecological opportunity, sexual selection and ecological versatility, can contribute to speciation in both sympatric and allopatric contexts (Seehausen 2000; Wagner et al. 2012). Allopatric speciation is well‐documented in cichlid lineages (Kornfield and Smith 2000; Kocher 2004), including in African rift lakes where fluctuations in lake levels have led to the separation and reconnection of habitat, promoting drift‐mediated differentiation and reproductive isolation of highly philopatric habitat specialists (Owen et al. 1990; Sturmbauer et al. 2001). Allopatric speciation has also been observed to have occurred in riverine systems, where species have evolved specialised phenotypes enabling them to occupy specific flow regimes (Markert et al. 2010; Alter et al. 2017; Kurata et al. 2022). Contributions of allopatric separation and local adaptation to speciation can be investigated using species that are distributed across physically divided habitats. The presence of distinct lineages in isolated habitats could highlight the role of allopatry, whereas evidence of ecomorphological divergence between separated populations may indicate a role of local adaptation in promoting reproductive isolation.

**FIGURE 1:**
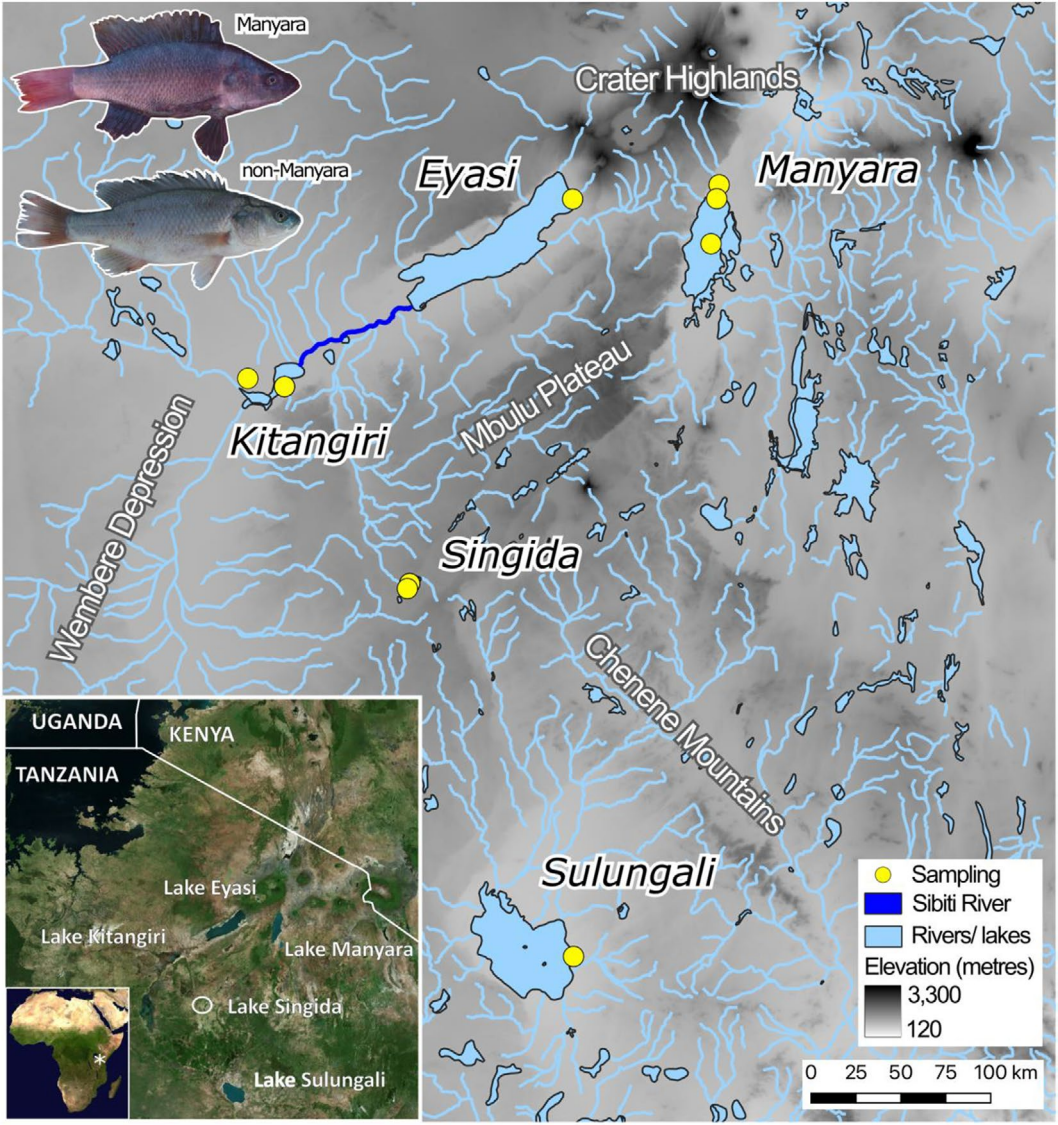
Locations where *Oreochromis amphimelas* was sampled. Sampling localities are indicated with yellow circles. Waterbodies and rivers shown in blue. Elevation is indicated using a grey scale. *O. amphimelas* individuals included from Manyara (photo MG) and Singida (photo JJD), with SL (snout‐caudal peduncle) measurements of 87 and 161 mm, respectively.

Here we study the potential for allopatric speciation in the soda lake cichlid *Oreochromis amphimelas* (Hilgendorf 1905), a species known from several lakes in the internal drainage basin of the Tanzanian rift system (Figure 1), which is south of Lakes Natron and Magadi. The species is a member of the cichlid tribe Oreochromini Dunz & Schliewen 2010, that includes both lacustrine and riverine species (Ford et al. 2019). Within this tribe, some lacustrine lineages have radiated to a limited extent (Ford et al. 2019), whereas most lineages have not (Ciezarek et al. 2024). The most striking *Oreochromis* radiation has taken place in soda Lake Natron, where three ecomorphologically divergent species are present: *Oreochromis alcalicus* (Hilgendorf 1905), *Oreochromis latilabris* Seegers & Tichy 1999 and *Oreochromis ndalalani* Seegers & Tichy 1999. These three species comprise a sister clade to *Oreochromis grahami* (Boulenger 1912), an endemic of soda Lake Magadi (Zaccara et al. 2014; Ford et al. 2016) and this phylogeographic pattern fits with evidence that Lakes Natron and Magadi once comprised a single palaeolake (Ford et al. 2016). These Lake Natron and Magadi species collectively are part of the subgenus *Oreochromis* (*Alcolapia*), which is a direct sister lineage to *O. amphimelas* (Ciezarek et al. 2024).

A study of genetic distances using nuclear markers (SNPs) revealed that the differentiation between the Lake Manyara and Lake Eyasi populations of *O. amphimelas* is greater than between the species of the subgenus of *Oreochromis* (*Alcolapia*) (Ford et al. 2015). It has also been observed that *O. amphimelas* populations from Lake Manyara show clear differences in mtDNA haplotypes from populations in other lakes (Eyasi, Kindai, Kitangiri, Singida and Sulungali—also known as Lake Sulunga and Bahi Swamp) (Morandus and Rumisha 2024). Collectively, these results provide evidence that there may be cryptic diversity in *O. amphimelas* . In this study, an integrative taxonomic approach was used to build on the existing evidence, including the combined use of genomic SNPs, mitochondrial DNA and morphological data to investigate genetic structuring and phenotypic diversity in *O. amphimelas* . Specific aims of the study were to: (1) investigate the phylogeography of *O. amphimelas* by sampling across the range of the species; (2) quantify morphological differences among lake populations and (3) test for evidence of species boundaries between genetically differentiated populations.

## 2 Materials and Methods

### 2.1 Focal Taxon *Oreochromis amphimelas*

*Oreochromis amphimelas* occurs in six soda lakes in the internal drainage basin of Tanzania (Trewavas 1983; Morandus and Rumisha 2024): Lakes Manyara, Eyasi, Kindai, Kitangiri, Singida and Sulungali (Shechonge et al. 2019). All lakes experience high UV exposure, evaporation rates and alkalinity (pH 8–12) (Lameck et al. 2024). *Oreochromis amphimelas* is the only tilapiine cichlid fish native to this basin (Trewavas 1983), but due to introductions of non‐native species, it now co‐occurs with *O. niloticus* in Lakes Sulungali, Singida, Kindai and Kitangiri and with *Oreochromis esculentus* in Lakes Singida, Kitangiri and Sulungali (Bwathondi 2002; Ford et al. 2019; Shechonge et al. 2019; Morandus and Rumisha 2024). Importantly, *O. amphimelas* is a locally fished species in the region. As such, it is possible that the *O. amphimelas* populations in Lakes Sulungali, Singida and Kindai may have been stocked (Bailey 1968; Trewavas and Fryer 1965; Shechonge et al. 2019), although reports of stocking do not preclude the possibility that the same taxon was already present. The six lakes have experienced varying degrees of connectivity throughout their history, associated with geographic features of the rift valley and fluctuations in lake levels (Foster et al. 1997; Olaka et al. 2010). The lakes containing *O. amphimelas* are separated into three endorheic sub‐basins of the internal drainage basin: (i) Lake Manyara, (ii) Lakes Eyasi, Kitangiri, Kindai and Singida and (iii) Lake Sulungali (Ring et al. 2005; Japan International Cooperation Agency 2008).

### 2.2 Sample Collection and DNA Extraction

Specimens of *O. amphimelas* were collected from five lakes; namely Manyara, Eyasi, Kitangiri, Singida and Sulungali (a total of nine sites; Figure 1, Table S1) in 2012, 2014, 2015 and 2017. Outgroup specimens of *O. niloticus* , *O. esculentus* and *Oreochromis (Alcolapia) alcalicus* were also included. Specimens were collected from artisanal fishers or by researchers using seine nets. Fish were euthanised with MS222 or clove oil. Genetic samples (fin clips) were stored in 95% ethanol, whereas whole specimens were also preserved in ethanol.

### 2.3 Reduced Representation Sequencing

Restriction site‐associated sequencing was used to derive genome‐wide SNP data from the four *Oreochromi*s cichlid species for a total of 96 samples; this comprised *O. amphimelas* from Lakes Eyasi (*n* = 8), Kitangiri (*n* = 10), Manyara (*n* = 49), Singida (*n* = 10) and Sulungali (*n* = 10) (Table S1) and outgroups *O. niloticus* (*n* = 3), *O. esculentus* (*n* = 2) and *O. alcalicus* (*n* = 4). DNA was extracted from fin clips using the DNeasy Blood and Tissue kit (Qiagen, Hilden, Germany). Library preparation followed the double‐digest RAD sequencing methods (ddRADseq) of Peterson et al. (2012). Standardised DNA (250 ng) was fragmented using the EcoRI and Mspl restriction enzymes and ligated to the barcoded adapters (Eurofins Scientific, Brussels, Belgium). Target fragments were then amplified using a 25‐cycle PCR, replicated four times. PCR products from the four amplifications were pooled and cleaned using a DNA Clean & Concentrator kit (Zymo Research, CA). Fragments in the range 200–500 bp were then extracted from a 2% TAE agarose gel, containing the PCRBIO III (PCRBiosystems, London, UK) and the E‐Gel 50 bp ladders (Invitrogen, MA), using the QIAquick Gel Extraction Kit (Qiagen, Hilden, Germany). The resulting library was quantified using the NEBNext Library Quant Kit (New England Biolabs, MA) in a PCRmax Eco48 thermocycler (Cole Parmer, IL). The final concentration was 9.96 nM (efficiency = 100 ± 1.91%, *r* ^2^ = 0.997). The library was sequenced using NextSeq 500/550 v.2.5 kits (Illumina, CA) at the University of Bristol Genomics Facility.

### 2.4 SNP Data: Genotyping and Filtering

Cutadapt v.2.0 (Martin 2011) was used to demultiplex the sequence indexes and trim barcodes, adapters and PCR indexes. Sequencing reads were aligned to the *O. niloticus* reference genome (GCF_001858045.2) using bwa v.0.7.17 (Li and Durbin 2009). Variant calling was performed on the resulting BAM files using freeBayes v1.3.7 (Garrison and Marth 2012). Following variant calling, 22 individuals were removed due to poor quality data, leaving 74 individuals with data for more than 60% of SNPs. Filtering for indels and SNPs with a maximum missingness of 0.5 and a minor‐allele count of 2 was applied using VCFtools v.0.1.13 (Danecek et al. 2011) and PLINK v.1.07 (Purcell et al. 2007). After filtering, the data comprised samples of 74 *O. amphimelas* from Lakes Eyasi (*n* = 6), Kitangiri (*n* = 8), Manyara (*n* = 35), Singida (*n* = 7) and Sulungali (*n* = 10) (Table S1) along with outgroups *O. niloticus* (*n* = 2), *O. esculentus* (*n* = 2) and *O. alcalicus* (*n* = 4).

We partitioned these data into four separate datasets. These included all species (Dataset 1; *n* = 74) and all *O. amphimelas* (Dataset 2; *n* = 66). Additionally, due to the greater number of individuals from Manyara and the potential bias caused by unequal sample sizes of different populations, we generated a randomly subsampled dataset to include an equal number of individuals for each *O. amphimelas* lake population (*n* = 4). These data, filtered only after subsampling, comprised either all species (Dataset 3; *n* = 27) or all *O. amphimelas* (Dataset 4; *n* = 20). Each of these four datasets was also filtered for linkage disequilibrium (LD) to investigate its effects and to meet the assumptions of STRUCTURE (Pritchard et al. 2000). Filtering was performed by calculating *r* ^2^ between every SNP pair within a sliding window of 500 kb and retaining one representative for each pair with an *r* ^2^ above 50%. This distance, derived from Ford et al. (2015), is cited as being the distance at which LD reaches background levels in species of the *Oreochromis* subgenus, *Alcolapia*, the sister clade of *O. amphimelas* (Ford et al. 2019; Ciezarek et al. 2024). This pairwise approach removed around half the variants in each dataset. However, as the relationships between each population were mostly unchanged by LD filtering, we used the non‐LD filtered dataset for all downstream analyses unless otherwise specified (McKinney et al. 2017).

### 2.5 SNP Data: Genetic Diversity and Genetic Differentiation

Conversion from VCF to data formats required for population genetic analyses was performed using PGDSpider v.3.0.0.0 (Lischer and Excoffier 2012). *F*_ST_ values (Nei 1973) were calculated using the package adegenet v.2.1.10 (Jombart 2008) in R v.4.3 (R Core Team 2019). Pairwise *F*_ST_ values and confidence intervals were also calculated for all loci per population in HIERFSTAT (Goudet 2005), bootstrapping over loci with 10,000 replicates. Nucleotide diversity (*π*), expected heterozygosity (*H*_E_) and observed heterozygosity (*H*_O_) were calculated in Stacks v.2.68 (Catchen et al. 2013).

### 2.6 SNP Data: Population Genetic Structure

Principal Component Analyses (PCA) were performed on each of the four datasets using adegenet v.2.1.0 (Jombart 2008). STRUCTURE v.2.3.4 (Pritchard et al. 2000) was run on Datasets 1 and 2 (filtered for LD) with 200,000 Markov‐Chain Monte Carlo (MCMC) runs and a burn‐in of 50,000 generations. Each run was repeated 10 times using a different starting seed. Runs were performed from *K* = 1 to *K* = 8. MCMC convergence was verified by comparing parameter states after the burn‐in and at the end of the run. The optimal number of clusters was inferred through the Evanno method (Evanno et al. 2005) using STRUCTURE Harvester (Earl and von Holdt 2012). The consensus result from each run was aggregated using CLUMPP v.1.1.2 with the full search method (Jakobsson and Rosenberg 2007).

### 2.7 SNP Data: Species Delimitation Analyses Using the Yule‐Skyline Collapse Model in SPEEDEMON

To quantitatively assess whether the genetic divergence between the Lake Manyara *O. amphimelas* population and other *O. amphimelas* populations can be considered to be species‐level, species delimitation analysis was performed using the package SPEEDEMON v.1.1 (Douglas and Bouckaert 2022) implemented in BEAST2 v.2.7.6 (Bouckaert et al. 2014). SPEEDEMON combines the collapse model (Jones et al. 2015) with the Yule‐skyline model (Bouckaert 2022) to create the ‘Yule‐skyline collapse’ model (Douglas and Bouckaert 2022). This model integrates birth rates analytically, which means that these parameters do not need to be estimated; however, the user needs to provide a collapse weight (epsilon), which is used as a threshold (which represents ancestral species time) below which samples are collapsed into a single species. For this analysis, Dataset 1 was subsampled to include the four *O. amphimelas* individuals from each lake population with the least missing data and all outgroup samples (*n* = 28); this subsampled Dataset 1 was then further filtered to include only sites with no missing data, a minor allele frequency of 0.05 and filtered for LD (with an *r* ^2^ value of 0.50 for each 1000 kb region) producing an alignment with 469 sites. Epsilon values of 10^−3^ and 10^−4^ were used, representing a range of effective values as tested in Douglas and Bouckaert (2022), and all other settings were left as default. Chains were run for 400 million generations, checked for convergence in Tracer v.1.7.2 (Rambaut et al. 2018) and analysed using the ClusterTreeSetAnalyser tool in SPEEDEMON.

### 2.8 SNP Data: Sliding Window Analysis

To assess differentiation across the genome, we calculated *F*_ST_ in windows between *O. amphimelas* populations (Dataset 2) using reference genome windows of 500 kb and steps of 20 kb with a minimum SNP per window threshold of 20. Including a minimum SNP threshold prevents elevated values due to low SNP density. Samples from Eyasi, Kitangiri and Singida were considered a single panmictic population for the analysis, based on the results of *a posterior* analysis. *F*_ST_ was calculated using parseVCFs.py and popgenWindows.py (https://github.com/simonhmartin/genomics_general/tree/master).

### 2.9 Mitochondrial DNA Sequence Data and Analyses

The complete mitochondrial control region (874 bp) was amplified following Day et al. (2007) using the primers LProF TDK‐D, SC‐DL and TDK‐DHG. The PCR products were cleaned using microCLEAN (Microzone, Stourbridge, UK); cycle sequenced using standard protocols; and analysed using an ABI13730xl sequencer. Sequences were edited using Sequencher v.4.8 (Gene Codes Corporation, Ann Arbor, MI) and manually aligned using SeAl v.2.0 (Rambaut 2002). The resulting nexus file was used for downstream analysis. IQ‐TREE v.2.4.0 (Minh et al. 2020) was used for phylogenetic inference using Maximum Likelihood on a subset of the full mtDNA dataset. Model selection was performed using the inbuilt ModelFinder Plus (Kalyaanamoorthy et al. 2017) and support was evaluated with 10,000 ultra‐fast bootstrap 2 iterations (Thi Hoang et al. 2017). The analysed data included 53 *O. amphimelas* individuals from Lakes Eyasi (*n* = 10), Kitangiri (*n* = 13), Manyara (*n* = 21) and Singida (*n* = 9).

### 2.10 Morphological Data Generation

Specimens that exhibited warping that could not be manipulated into a natural position or were too badly damaged were excluded from morphometric analysis. This resulted in the selection of a total of 121 ethanol‐preserved specimens of *O. amphimelas* for analysis from Lakes Eyasi (*n* = 12), Kitangiri (*n* = 15), Manyara (*n* = 59), Singida (*n* = 12) and Sulungali (*n* = 23). All specimens, except for the holotype, were collected and preserved between 2012 and 2017. Each specimen was photographed on the left‐hand side using a tripod‐mounted Canon EOS 20D DS126061 camera and Canon Macro lens EF 100 mm 1:2.8 USM. Specimens were pinned and photographed alongside a 30 cm ruler. For reference, a measurement of standard length (SL) was taken using digital callipers. In most cases, external sexing of individuals could not be achieved and was instead carried out by dissection. Of the 121 specimens, 71 were sexed (Table S1).

### 2.11 Landmarking, Data Validation and Geometric Morphometric Analyses

Geometric morphometric analyses were based on 14 homologous landmarks (Figure S1), selected from Ford et al. (2016). TPS files were generated from photographs using tpsUtil v.1.76 (Rohlf 2015), and landmarks were digitised using tpsDig v.2.31 (Rohlf 2015). The consistency of landmarking was tested by randomly selecting twenty specimens using the *sample.int* function in R v.4.3 and re‐landmarking these specimens in a blind test. We assessed measurement error using Procrustes ANOVA, which revealed no significant effect of replication (*F*_1_,_1115_ = 0.005, *p* = 0.949) and no significant specimen × replication interaction (*F*_1_,_1115_ = 0.0001, *p* = 0.994). These results indicate high repeatability of our landmarking protocol, with no evidence of systematic measurement bias between landmarking sessions or specimen‐specific landmarking difficulties. Therefore, no repeats were conducted for downstream analyses.

Principal Component Analysis was carried out in geomorph v.4.0.10 (Baken et al. 2021; Adams et al. 2025). Landmark coordinate data was imported, and Generalised Procrustes Analysis was performed using the function gpagen in geomorph to scale and optimally rotate landmark data. Procrustes aligned coordinates were checked for outliers using the function plotOutliers in geomorph, before the function gm.prcomp in geomorph was used to perform PCA on aligned coordinates. Plots of the PCA results were produced in ggplot2 (Wickham 2016). PCA was also repeated on aligned coordinates in MorphoJ 1.06d (Klingenberg 2011) to produce transformation grids (Figure S7), enabling the visualisation of morphological changes associated with each PC axis.

### 2.12 Meristic Data and Analyses

Counts of eight meristic characters were taken for each of the 121 specimens included in the geometric morphometric analyses (Tables S1 and S2) following Stauffer Jr. et al. (2008). These characters were the number of: (1) lateral line scales, (2) dorsal fin spines, (3) dorsal fin rays, (4) anal fin spines, (5) anal fin rays, (6) pelvic fin spines, (7) pelvic fin rays and (8) pectoral fin rays. Kruskal–Wallis tests were used to assess whether meristic counts differed significantly among populations using the dunn.test package in R v.4.3 (Dinno 2024). *Post hoc* pairwise comparisons were conducted using Dunn's tests, with Bonferroni correcting for multiple testing, using the dunn.test package. To evaluate differences between the sexes, Wilcoxon tests were performed using the wilcox.test function (stats package, R v.4.3).

## 3 Results

### 3.1 SNP Data and Genetic Diversity

A total of 17,180,030 reads were sequenced across all samples, totalling 5.1 Gbp. The proportion of reads with > Q30 was 58.73% and 44.86% for forward and reverse reads, respectively. The number of reads sequenced per individual ranged from 3203 to 321,470. Dataset 1, which contained 74 individuals across the four focal species, comprised 142,107 variants and, after filtering for LD, 57,537 sites. Dataset 2, containing 66 *O. amphimelas* individuals from the five sampled lakes, comprised 86,164 variants (46,578 after filtering for LD). To compare the genetic diversity among populations, we used two subsampled datasets. Dataset 3, which contained 27 individuals across the four focal species, comprised 66,409 variants and, after filtering for LD, 22,172 sites (Table 1). Dataset 4, containing 20 *O. amphimelas* individuals from the five sampled lakes, comprised 29,178 variants and, after filtering for LD, 15,813 variants.

**TABLE 1:** Population genetic statistics for populations in this study, using the subsampled ddRAD derived SNP datasets (Dataset 3, *n* = 27).

Group	*n*	*H*_O_	*H*_E_	*π*
*Oreochromis amphimelas* (Lake Eyasi)	4	0.01561	0.05245	0.06317
*O. amphimelas* (Lake Kitangiri)	4	0.01296	0.04769	0.05724
*O. amphimelas* (Lake Sulungali)	4	0.01771	0.05487	0.06464
*O. amphimelas* (Lake Singida)	4	0.01679	0.05852	0.06966
*O. amphimelas* (Lake Manyara)	4	0.01414	0.04241	0.04974
*Oreochromis esculentus*	2	0.01155	0.02162	0.03021
*Oreochromis niloticus*	2	0.01145	0.04159	0.05616
*Oreochromis alcalicus*	3	0.01463	0.05393	0.06712

Across the four species sampled, *O. alcalicus* had the highest nucleotide diversity and *O. esculentus* had the lowest (Dataset 3; Table 1). Meanwhile, across the *O. amphimelas* populations sampled, Lake Manyara had the lowest nucleotide diversity and expected heterozygosity (Dataset 3; Table 1).

### 3.2 Population Genetic Structure

**FIGURE 2:**
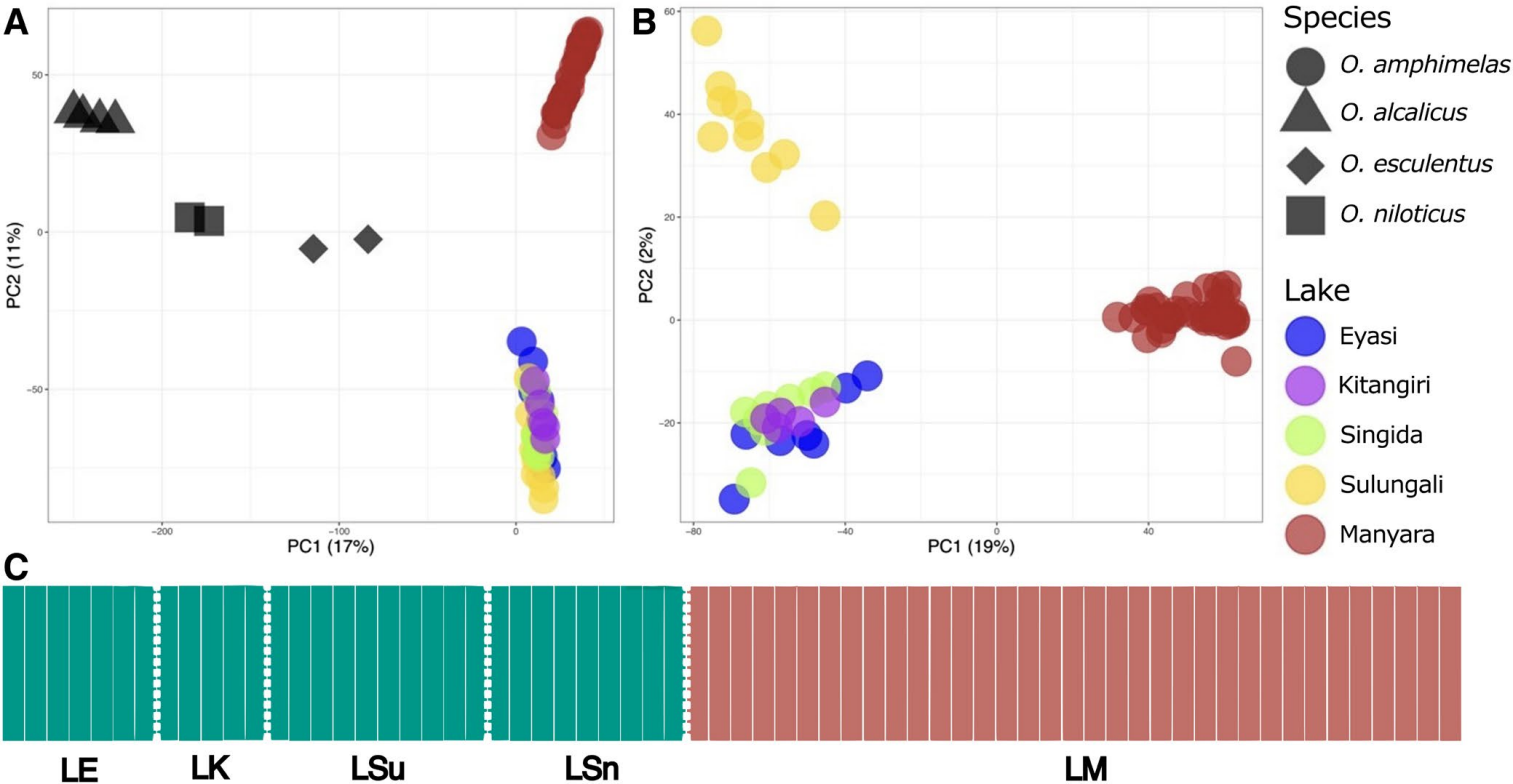
Genetic clustering of sampled individuals, based on the reduced representation whole genome data. Shown are PCA plots of the first two Principal Component axes for (A) Dataset 1 and (B) Dataset 2. Each point represents an individual sample; shapes indicate the species; colours indicate the lake of origin of *O. amphimelas* samples. The percentage of measured variation explained by each axis is shown in brackets next to the axis titles. (C) Structure plot from Dataset 2 (LD‐filtered) showing the assignment of each *O. amphimelas* individual to *K* = 2 clusters. Each vertical bar represents an individual and each group is separated by dashed white lines. Group labels are shown at the bottom, LE, Lake Eyasi; LK, Lake Kitangiri; LM, Lake Manyara; LSn, Lake Singida; LSu, Lake Sulungali. For descriptions of datasets, see Materials and Methods, SNP data: *Genotyping and filtering*.

In comparisons of all four *Oreochromis* species (Dataset 1), there was strong differentiation between species, with all interspecific comparisons having *F*_ST_ > 0.5 (Table 2). There was interspecific clustering along the first principal component axis, which separated *O. amphimelas* from the other three species. The second principal component separated Lake Manyara *O. amphimelas* from *O. amphimelas* individuals in other lakes (Figure 2A). We found no clear evidence of hybrid individuals between *O. amphimelas* and the non‐native species *O. niloticus* and *O. esculentus* . In the analysis of the subsampled dataset including all four species (Dataset 3), the clear structure among species was again present (Figure S2c).

**TABLE 2:** Median pairwise *F*_ST_ values, calculated in HIERFSTAT with 10,000 bootstrap replicates, with 95% confidence intervals in parentheses using Dataset 1.

	Esc	Nil	LE	LK	LM	LSn	LSu
Alc	0.554 (0.547–0.561)	0.633 (0.627–0.638)	0.672 (0.667–0.676)	0.678 (0.673–0.683)	0.713 (0.710–0.717)	0.686 (0.681–0.690)	0.704 (0.700–0.708)
Esc	—	0.542 (0.532–0.551)	0.612 (0.606–0.619)	0.645 (0.639–0.652)	0.633 (0.628–0.638)	0.612 (0.606–0.618)	0.633 (0.628–0.639)
Nil		—	0.705 (0.701–0.710)	0.722 (0.717–0.726)	0.733 (0.729–0.736)	0.715 (0.711–0.720)	0.732 (0.728–0.736)
LE			—	−0.005 (−0.012–0.002)	0.346 (0.338–0.353)	0.012 (0.006–0.017)	0.075 (0.069–0.081)
LK				—	0.328 (0.320–0.335)	0.010 (0.004–0.017)	0.071 (0.064–0.078)
LM					—	0.347 (0.340–0.354)	0.372 (0.365–0.379)
LSn						—	0.068 (0.063–0.073)

*Note:* Population/species abbreviations are as follows: Alc, *O. alcalicus*; Esc, *O. esculentus* ; Nil, *O. niloticus* ; Grey shading denotes *O. amphimelas* ; LE, Lake Eyasi; LK, Lake Kitangiri; LM, Lake Manyara; LSn, Lake Singida; LSu, Lake Sulungali. Comparisons within *O. amphimelas* are shown by shaded cells.

Within *O. amphimelas* (Dataset 2), the Lake Manyara population was highly differentiated from all other populations, with all population comparisons having *F*_ST_ > 0.3 (Table 2; Figure S3). In PCA, the first axis, PC1, separated Lake Manyara from the other lakes and PC2 separated Lake Sulungali from Lakes Eyasi, Kitangiri and Singida (Figure 2B). This result was also observed in the subsampled dataset containing only the five *O. amphimelas* populations (Dataset 4; Figure S2e). STRUCTURE analysis of the five *O. amphimelas* populations (Dataset 2) identified *K* = 2 as optimal (Evanno method), assigning Lake Manyara and non‐Manyara *O. amphimelas* individuals to different clusters, with no clear evidence of admixture between these two groups (Figure 2C).

### 3.3 Species Delimitation With SPEEDEMON

At *ε* = 0.001 and *ε* = 0.0001, using the alignment produced from a further‐filtered and subsampled Dataset 1 (described above), the best supported hypothesis included five species with the following topology at *ε* = 0.001 (92.44%) and *ε* = 0.0001 (95.91%):

((*Oreochromis alcalicus*, ( *Oreochromis esculentus* , *Oreochromis niloticus* )), (Manyara *Oreochromis amphimelas* , non‐Lake Manyara *Oreochromis amphimelas* ));

In both species delimitation analyses at *ε* = 0.001 and *ε* = 0.0001, Manyara *O. amphimelas* and non‐Manyara *O. amphimelas* clusters each received 100% support; but there was no support for a single combined *O. amphimelas* cluster (Table 3).

**TABLE 3:** SPEEDEMON species delimitation results at different thresholds to collapse species for the 95% credible set.

Lineage	Support at *ε* = 0.001 (%)	Support at *ε* = 0.0001 (%)
Non‐Manyara *Oreochromis amphimelas*	100	100
Manyara *O. amphimelas*	100	100
*Oreochromis alcalicus*	100	100
*Oreochromis niloticus*	97.83	99.47
*Oreochromis esculentus*	97.83	99.47
*O. esculentus* + *O. niloticus*	2.17	0.53

### 3.4 Sliding Window Analysis

**FIGURE 3:**
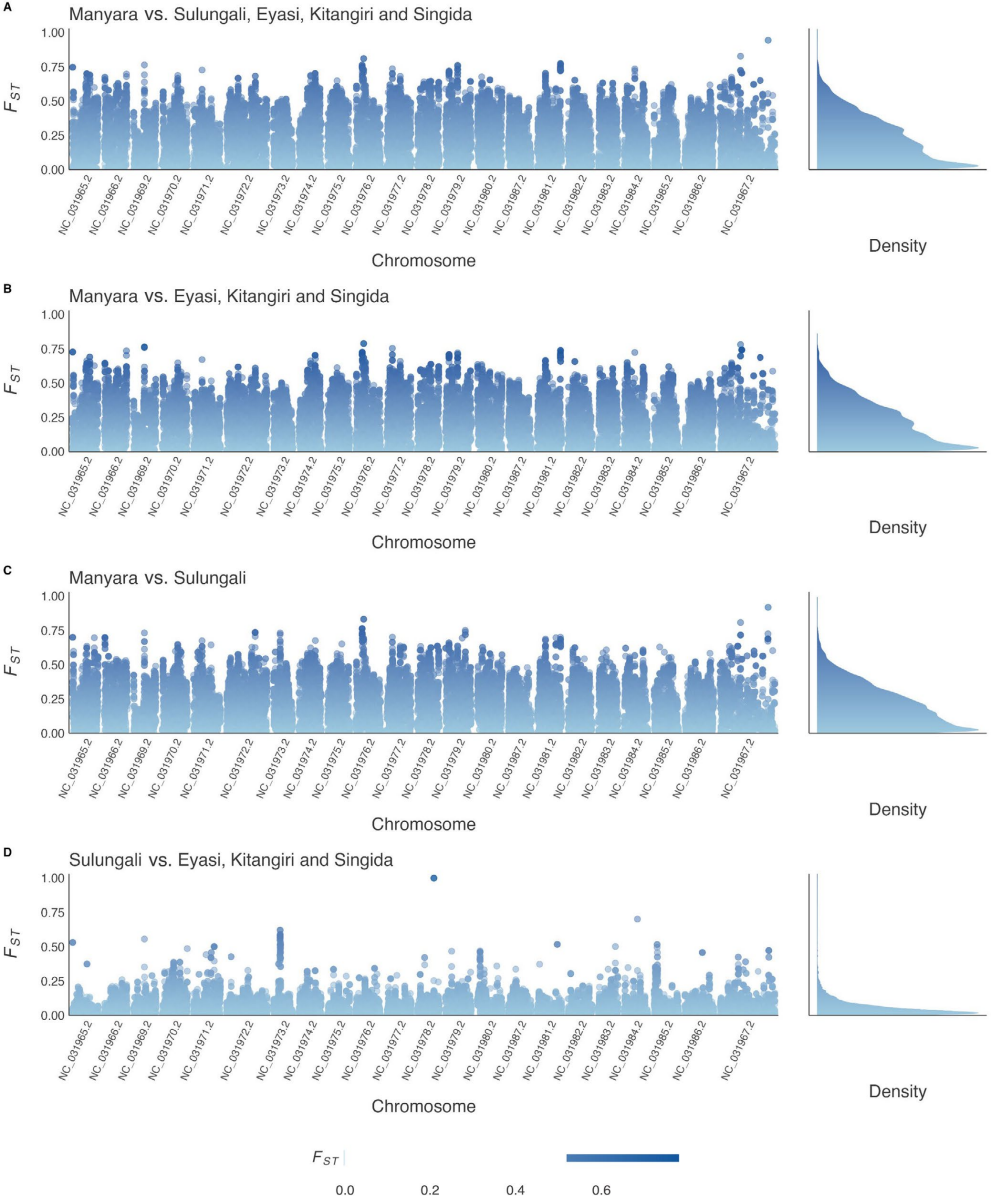
Genome‐wide sliding window analysis of relative divergence for pairwise population comparisons and the density distribution of *F*_ST_. (A) Manyara versus Sulungali, Eyasi, Kitangiri and Singida (B) Manyara versus Eyasi, Kitangiri and Singida (C) Manyara versus Sulungali (D) Sulungali versus Eyasi, Kitangiri and Singida. Window size is 500 kb with a slide of 20 kb and a minimum SNP number of 20.

Comparisons of *O. amphimelas* populations (Dataset 2) that included Lake Manyara showed a high *F*_ST_ across the whole genome (Figure 3A–C), with some highly divergent genomic windows characterised by high *F*_ST_ peaks. These included regions on chromosomes NC_031969 and NC_031976 not found in the Lake Sulungali versus Lake Eyasi, Kitangiri, Singida comparison (Figure 3D). There was an overall lower level of windowed *F*_ST_ between Lake Sulungali and the other non‐Manyara lakes (Figure 3D), this comparison revealed some highly divergent genomic regions on chromosomes NC_031973, NC_031980 and NC_031985.

### 3.5 Mitochondrial DNA Sequences

The Maximum Likelihood tree generated by IQ‐TREE identified a deep, well‐supported (ultra‐fast bootstrap values of 100) split between Lake Manyara *O. amphimelas* individuals and non‐Manyara *O. amphimelas* (Figure S4).

### 3.6 Analyses of Morphological Data

**FIGURE 4:**
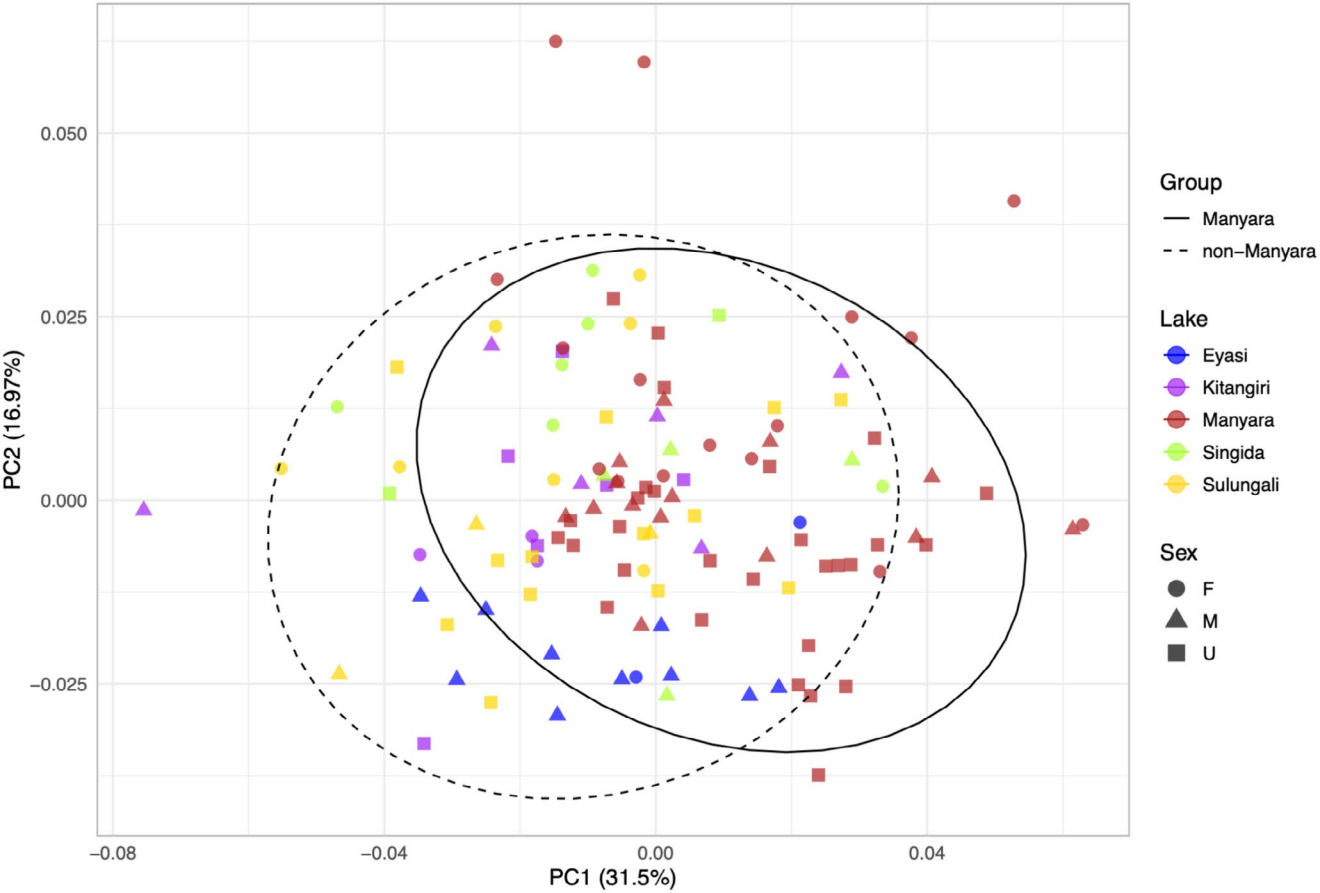
Principal Component Analysis illustrating morphospace of the five *Oreochromis amphimelas* populations, based on geometric morphometric data from 14 homologous landmarks. Solid ellipses represent 95% confidence intervals for the ‘Manyara’ group, and dashed ellipses represent 95% confidence intervals for the ‘non‐Manyara’ group.

Across the five lake populations, none occupied a distinct region of morphospace as defined using the first four principal components (Figure 4; Figure S5). In total, these first four PC axes described 68.1% of total shape variation, corresponding to 31.5%, 17%, 10.7% and 8.9%, respectively. PC1 corresponded to body depth, as well as the relative distance between the posterior insertion of the pectoral fin and the anterior insertion of the anal fin (Figure S6). PC2 was associated with head length relative to body length (Figure S6). PC3 corresponded to the relative length of the anal fin, and PC4 corresponded to the relative position of the caudal fin ventral base (Figure S6). There was also no evidence of males and females segregating in morphospace (Figure 4; Figure S5). These results were qualitatively unchanged when three possible outliers were removed (high PC2 values; individuals AF‐15‐026‐34, AF‐12‐02‐05 and AF‐12‐02‐04).

In meristic variables, there was no variation among the sampled specimens in the number of anal fin spines (always three), pelvic fin spines (always one) and pelvic fin rays (always five) (Table S2). In global analyses (Kruskal–Wallis tests), there were differences among populations in lateral line scales (*χ* ^2^ = 32.59, df = 4, *p* < 0.001), dorsal fin spines (*χ* ^2^ = 20.18, df = 4, *p* < 0.001), anal fin rays (*χ* ^2^ = 12.29, df = 4, *p* = 0.02) and pectoral fin rays (*χ* ^2^ = 68.20, df = 4, *p* < 0.001). There were no differences among the sampled populations in the number of dorsal fin rays (*χ* ^2^ = 8.59, df = 4, *p* = 0.07). Post hoc analyses (Dunn's tests) showed that all population pairs differed in at least one meristic variable, with the exception of the comparison of Lake Eyasi and Lake Kitangiri populations (Table S3; Figure S7). There were no significant differences between the sexes in any meristic variables (Wilcoxon tests; females *n* = 34; males *n* = 37): lateral line scales (*W* = 528, *p* = 0.698), dorsal fin spines (*W* = 571, *p* = 0.450), dorsal fin rays (*W* = 639.5, *p* = 0.898), anal fin rays (*W* = 706, *p* = 0.334) and pectoral fin rays (*W* = 633, *p* = 0.611).

## 4 Discussion

### 4.1 Allopatric Divergence in *O. amphimelas* Associated With Landscape Structure

Our findings highlight two genetically distinct *O. amphimelas* lineages (Manyara and non‐Manyara), despite morphological conservatism. Thus, our results based on nuclear and mtDNA data are consistent with the findings of Morandus and Rumisha (2024). This genetic structuring of *O. amphimelas* , with no evidence of hybridization or ongoing gene flow between the Manyara and non‐Manyara populations, closely mirrors the geomorphology of the region. The Lake Manyara population occupies the North–South pointing Natron‐Manyara‐Balangida rift (Macheyeki et al. 2008), whereas the non‐Manyara populations occupy the North‐East pointing Eyasi‐Wembere rift (Foster et al. 1997; Ring et al. 2005). The highland region between these rifts comprises the predominantly unfaulted Mbulu plateau highland region and the crater Highlands (Barboni 2014; Foster et al. 1997) (Figure 1). Lake Manyara, situated in the Natron‐Manyara‐Balangida rift, has therefore been somewhat isolated from other seasonal soda lakes since its formation (Trewavas 1983; Foster et al. 1997; Barboni 2014), at least by the Middle Pleistocene (Ring et al. 2005) (Figure 1). Nonetheless, dispersal of fish may have been possible between the two rift basins during the Late Pleistocene and Early Holocene highstands, via affluent rivers and outlet tributaries southwest of Lake Manyara (Barker 1992; Casanova and Hillaire‐Marcel 1992).

After subsampling to a standard sample size, the Manyara population was found to have relatively low genomic diversity relative to other lakes sampled (corroborating Morandus and Rumisha 2024). This is perhaps surprising, given the historically larger area of the lake. During past highstands, it has been estimated to have been over four times larger than present, with the eastern coast of the lake extended 40 km further east than at present (Ring et al. 2005). It is plausible that the low genomic diversity reflects the relative isolation of Manyara, and also potentially a loss of genetic diversity following drought‐linked population decline (Bayona 2006).

### 4.2 Connectivity Between Non‐Manyara *O. amphimelas* Populations

Our results show no clear genetic structure among the *O. amphimelas* populations from the non‐Manyara lakes Kitangiri, Eyasi and Singida, which are a chain of lakes along the Eyasi–Wembere rift (Trewavas and Fryer 1965; Trewavas 1983; Barboni 2014) (Figure 1). In its formative pre‐Pleistocene years, Lake Eyasi included Lake Kitangiri (Verniers 1997), but now these lakes are connected by the Sibiti River. More broadly, these lakes are in the same internal drainage sub‐basin as the endorheic Lake Singida (Trewavas 1983; Japan International Cooperation Agency 2008), and plausibly Singida has historically had riverine connections to Lake Eyasi and Lake Kitangiri during historic high lake stands, through which fish could have naturally moved. However, the presence of Lake Singida *O. amphimelas* , and the genetic similarities with Lakes Eyasi and Kitangiri, are most parsimoniously explained by lake stocking practices during colonial times. Trewavas and Fryer (1965) remarked that Lake Singida ‘is known to have been stocked’ with *O. amphimelas* , whereas Bailey (1968) considered that *O. amphimelas* was ‘almost certainly introduced’ into Lake Singida, but did not further elaborate on any available evidence.

We found that the Lake Sulungali population diverged from other non‐Manyara lakes, based on hierarchical population structure analysis (Figure 2a,b). Lake Sulungali, also known as Bahi Swamp, is south of Lake Singida and has a distinct endorheic catchment in a sub‐basin that is separated from the lakes in the Eyasi–Wembere rift (Figure 1, Macheyeki et al. 2008; Japan International Cooperation Agency 2008). The genetic differences between Lake Sulungali and the three sampled lakes in the Eyasi–Wembere rift could indicate that the Lake Sulungali population is naturally occurring, rather than being introduced. The Lake Sulungali *O. amphimelas* population was recorded in 2002, coexisting with *O. esculentus* and *O. niloticus* (Bwathondi 2002), both of which are certainly introduced. The genetic differentiation we observed between Lake Sulungali *O. amphimelas* and those in the three Eyasi–Wembere rift lakes may have arisen through extreme genetic drift and/or selection (Wolf and Ellegren 2017), which may explain the apparent islands of genomic differentiation observed in comparisons between these populations (Figure 3D).

## 5 Systematic and Evolutionary Implications

The lack of admixture between the Lake Manyara and non‐Manyara populations of *O. amphimelas* (Figures 2c and 3) and evidence from our species delimitation analyses collectively indicate these are distinct evolutionary lineages that may plausibly represent distinct species. We found no evidence for diagnostic morphometric or meristic differences between these two major lineages of *O. amphimelas* ; although we did not investigate differences in gill rakers or dentition, which may reflect ecological niche differentiation or sexual selection between the two *O. amphimelas* lineages.

Our results confirm the absence of any radiations of *O. amphimelas* within lakes, which is notable given the size of the lakes and the absence of clear competitor lineages within them. Thus, it is possible that *O. amphimelas* is constrained in other aspects of ecological opportunity, such as the availability of predictable food resources across a range of distinct habitats. The soda lakes in the East African Rift Valley tend to be relatively shallow, turbid and have lake substrates comprising only muddy sediment. Thus, it is plausible that this relatively homogenous habitat has not enabled diversification. Notably, the sister lineage to *O. amphimelas* —the *Oreochromis* subgenus *Alcolapia*—that has radiated in Lake Natron has only done so in the potentially more heterogenous flowing clear‐water springs where they have diverged in both dietary ecology and trophic morphology (Ford et al. 2016).

Disruptive sexual selection is also hypothesised to be an important driver of rapid radiations (Kornfield and Smith 2000; Kraaijeveld et al. 2011), and specifically in cichlids (Seehausen and van Alphen 1999; Wagner et al. 2012). *Oreochromis amphimelas* primarily occurs in naturally turbid waters, and although breeding males have striking red and black breeding colours (Figure 1), it is possible that the turbid waters may have constrained their diversification within the soda lakes. Notably, sympatric *Oreochromis* (*Alcolapia*) species in the clear water Lake Natron springs exhibit strikingly different male breeding colours (Ford et al. 2016) and show assortative mating (Lawson et al. 2023).

## 6 Concluding Remarks

Our results show that while *O. amphimelas* is morphologically conserved, there is strong genetic structuring between the Manyara and non‐Manyara populations, indicating that these two lineages may represent cryptic species. This has important implications for its conservation. Currently, *O. amphimelas* is categorised as ‘Endangered’ by the International Union for the Conservation of Nature Red List, in part due to active artisanal fisheries that target the species in multiple lakes (Bayona 2006; IUCN 2006). Given that the type specimen of *O. amphimelas* is from Lake Manyara, formal recognition of the non‐Manyara populations as a second species in the *O. amphimelas* lineage would considerably reduce the known geographic range of *O. amphimelas* , and therefore potentially elevate the threat of extinction. Based on our results, we suggest that further genomic and transcriptomic evidence could be used to determine the extent of genomic differentiation between the *O. amphimelas* lineages, and to explore specific adaptations that populations of this species have evolved to overcome challenges they face in extreme soda lake conditions.

## Author Contributions

**Miranda B. Sherlock:** formal analysis (lead), investigation (lead), visualization (lead), writing – original draft (lead), writing – review and editing (lead). **Emily Phelps:** formal analysis (equal), investigation (equal), writing – review and editing (equal). **Kenji Yip Tong:** formal analysis (equal), investigation (equal), writing – review and editing (equal). **Ewan H. Bodenham:** formal analysis (equal), investigation (equal), writing – review and editing (equal). **Asilatu H. Shechonge:** resources (equal). **Antonia G. P. Ford:** writing – review and editing (equal). **George F. Turner:** resources (equal), writing – review and editing (equal). **Martin J. Genner:** conceptualization (equal), funding acquisition (equal), resources (equal), writing – review and editing (equal). **Julia J. Day:** conceptualization (equal), funding acquisition (equal), resources (equal), supervision (lead), writing – review and editing (equal).

## Ethics Statement

Samples were collected with permission from the Tanzania Commission for Science and Technology, under the following permits: 2012‐23‐NA‐2011‐182; 2014‐23‐NA‐2011‐182; 2014‐374‐ER‐2011‐103.

## Conflicts of Interest

The authors declare no conflicts of interest.

## Supporting information


**Data S1:** ece372054‐sup‐0001‐FigureS1‐S7.docx.

**Table S1:** Summary details of the samples analysed in the study.
**Table S2:** Summary statistics, including standard length, and the five meristic variables investigated among populations.
**Table S3:** Dunn's tests for differences of meristic counts among the five *O. amphimelas* populations; *p* values are Bonferonni corrected, *p* < 0.05 in bold.

## Data Availability

Illumina sequence data are deposited at the Sequence Read Archive under BioProject PRJNA1250140. SNP genotype, mtDNA and morphological data are available at: https://doi.org/10.5281/zenodo.16697566.
