# Supplementary material for: Genomic Data Reveals Cryptic Diversity in the Soda Lake Cichlid Oreochromis amphimelas
Source: Ecol Evol. 2025 Sep 11;15(9):e72054. doi: 10.1002/ece3.72054 (PMC12425602; doi:10.1002/ece3.72054)
Supplement: Supplementary file 1 — Data S1: ece372054‐sup‐0001‐FigureS1‐S7.docx. [file ECE3-15-e72054-s002.docx]

**Genomic data reveals cryptic diversity in the soda-lake cichlid *Oreochromis amphimelas***

Miranda B. Sherlock, Emily Phelps, Kenji Yip Tong, Ewan H. Bodenham, Asilatu Shechonge,

Antonia G.P. Ford, George F. Turner, Martin J. Genner, Julia J. Day

**Supplementary Figures**


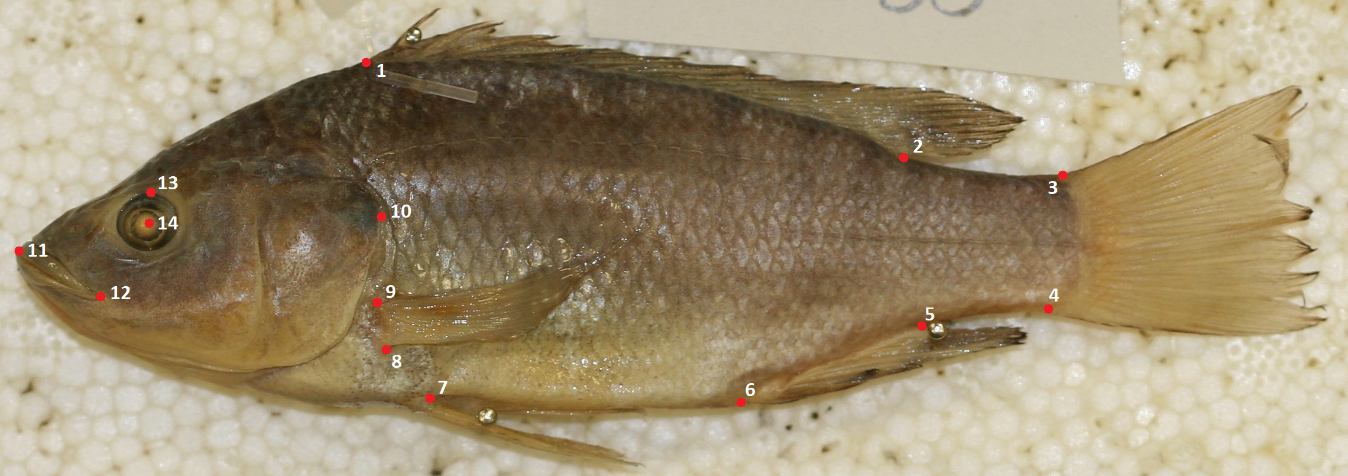


**Figure S1**. *Oreochromis amphimelas* specimen annotated with landmarks for geometric morphometrics. (1) Anterior insertion of dorsal fin, (2) Posterior insertion of dorsal fin, (3) Dorsal insertion of caudal fin, (4) Ventral base of caudal fin, (5) Posterior insertion of anal fin, (6) Anterior insertion of anal fin, (7) Anterior insertion of pelvic fin, (8) Lower insertion of pectoral fin, (9) Upper insertion of pectoral fin, (10) Posterior extremity of operculum, (11) Lip juncture (anterior snout tip), (12) Posterior point of mouth cleavage, (13) Dorsal margin of the eye, (14) Centre of the orbit.

A B

C D

E F

**Fig. S2**. PCA plots of the first two principal components for A) Dataset 1 (LD-filtered) B) Dataset 2 (LD-filtered), C) Dataset 3, D) Dataset 3 (LD-filtered), E) Dataset 4, F) Dataset 4 (LD-filtered). Each point represents a sample. Colours indicate the lake of origin of *O. amphimelas* samples, red = Manyara, blue = Eyasi, yellow = Sulungali, green = Singida, purple = Kitangiri. For descriptions of datasets, see Materials and Methods, SNP data: *Genotyping and filtering.*

A B


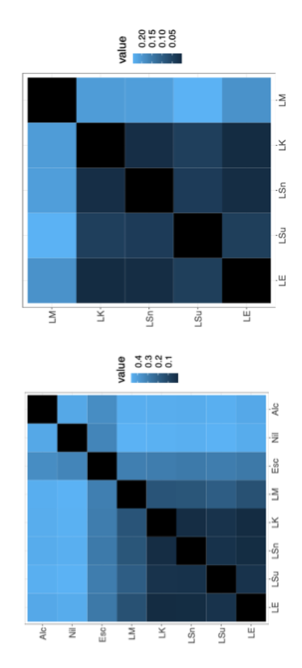

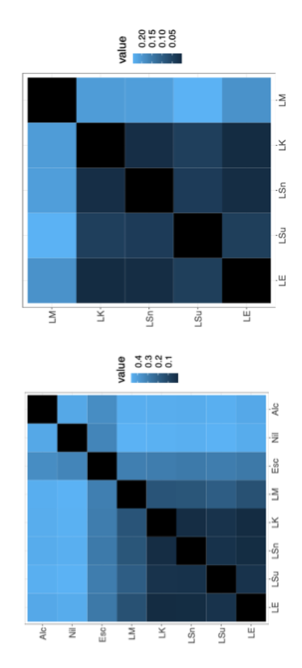


**Figure S3**. Heat maps showing pairwise *F*_ST_ between different cichlid species and populations: A. Dataset 1; B. Dataset 2. Colours show the values as indicated by the scales. Each heat map has its own scale, and the colour range is relative to the maximum value in each heat map. Population/ species abbreviations are as follows: Alc = *Orechromis* *(Alcolapia) alcalica*, Nil = *O. niloticus*, Esc = *O. esculentus*, LM = Lake Manyara, LK = Lake Kitangiri, LSn = Lake Singida, LSu = Lake Sulungali, LE = Lake Eyasi.


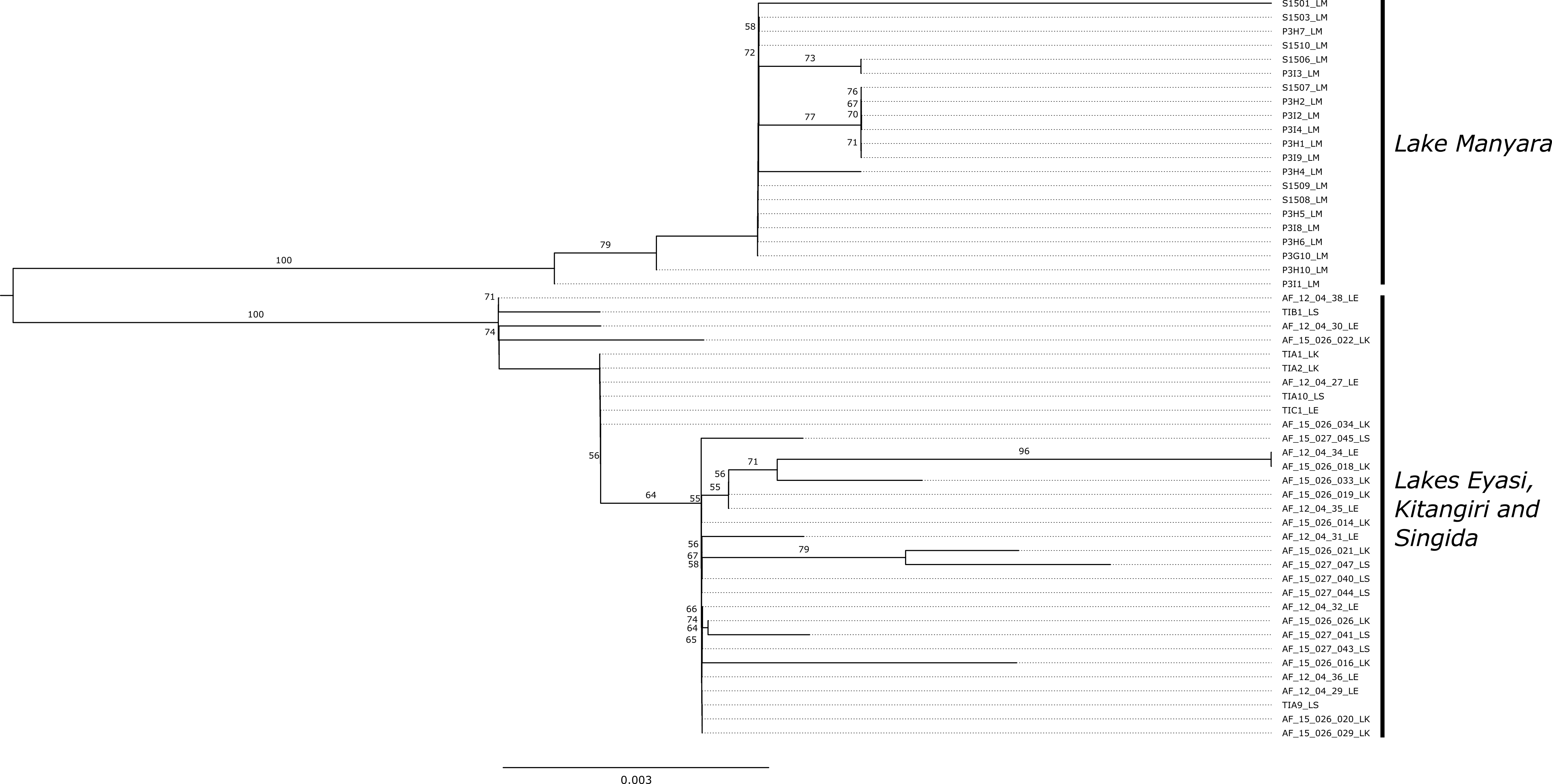


**Figure S4**. IQTREE2 Maximum Likelihood tree of *Oreochromis amphimelas* individuals using the complete mitochondrial control region (872 bp). Branch support values are ultra-fast bootstrap 2 values (support less than 0.5 not shown).

A

B

**Figure S5**. Principal Component Analysis (PCA) biplot showing geometric morphometric shape variation in *Oreochromis amphimelas* for A) PC1 vs. PC3 and B) PC1 vs. PC4. Solid ellipses represent 95% confidence intervals for the ‘Manyara’ group and dashed ellipses represent 95% confidence intervals for the ‘non-Manyara’ group.


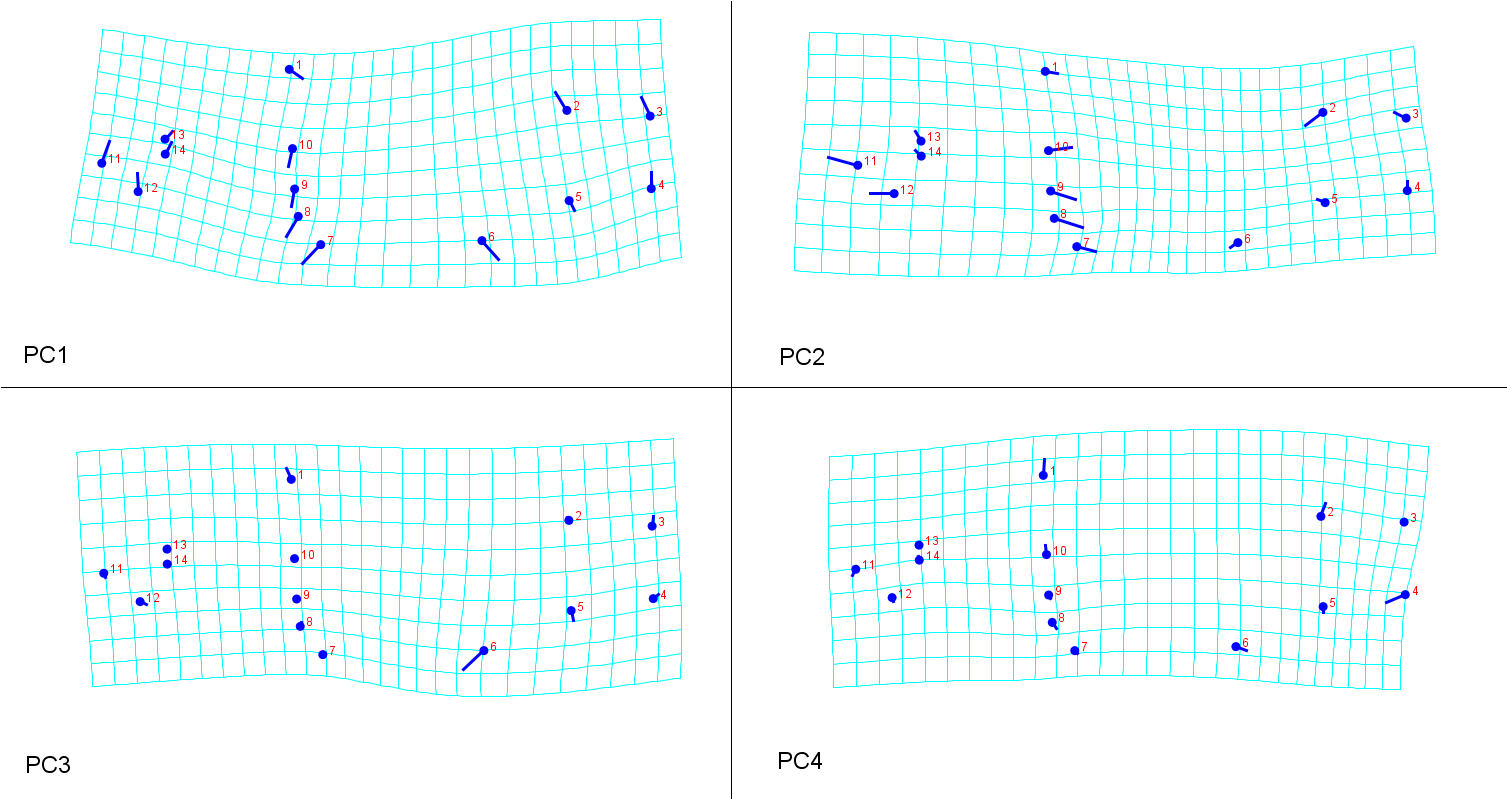


**Figure S6**. Transformation grids showing shape change of *Oreochromis amphimelas* individuals associated with PC1-4 for the complete dataset.

A B


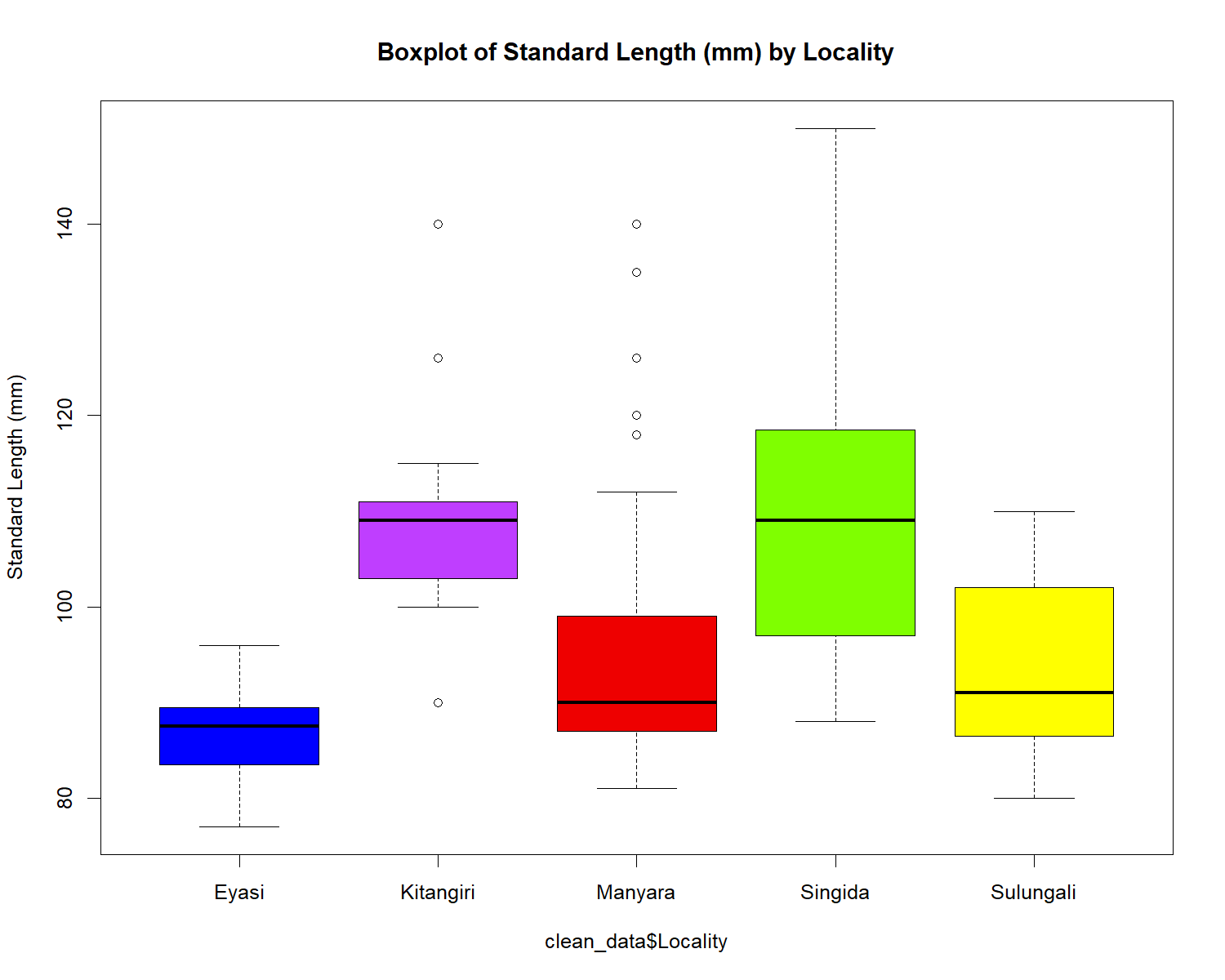

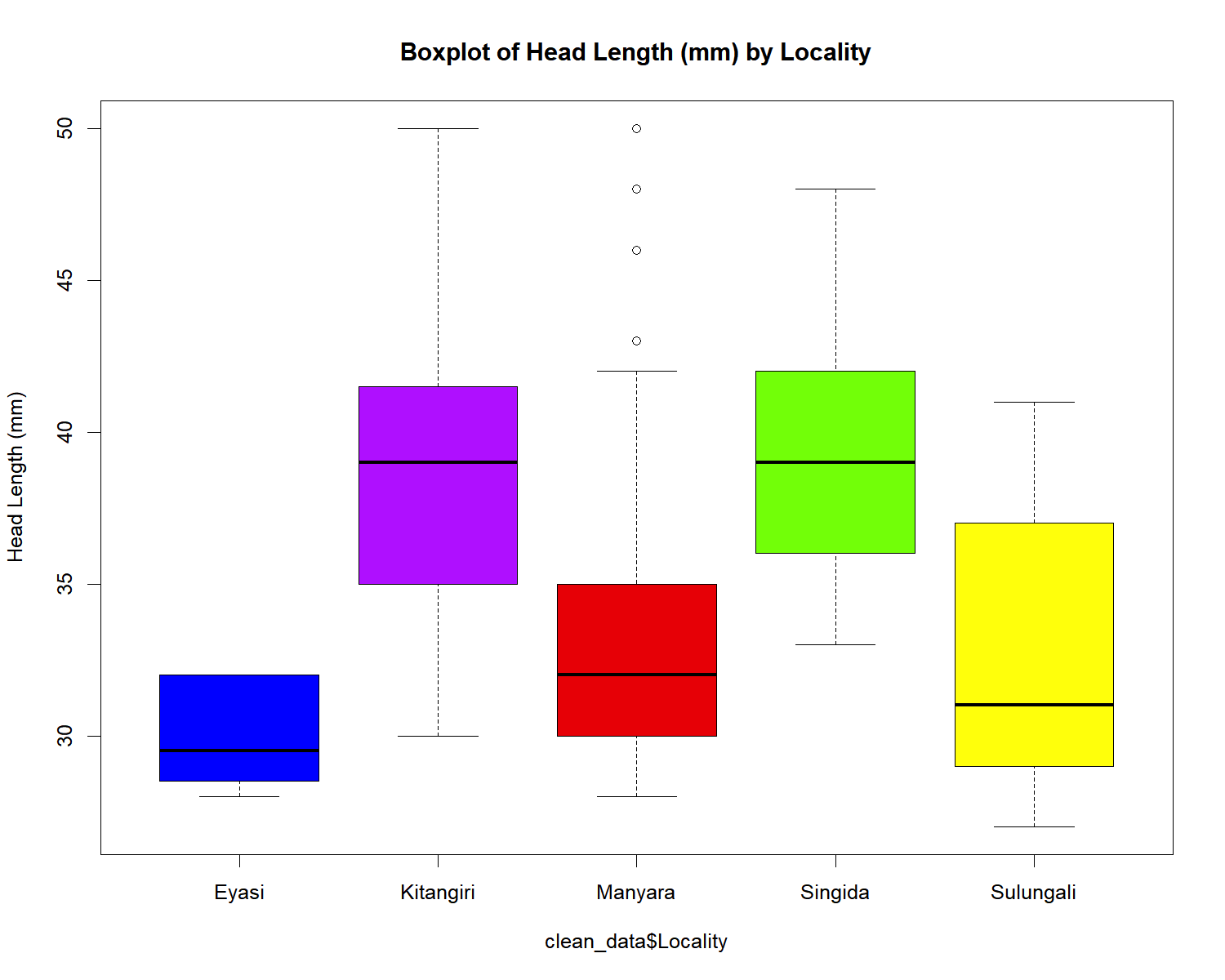


C D


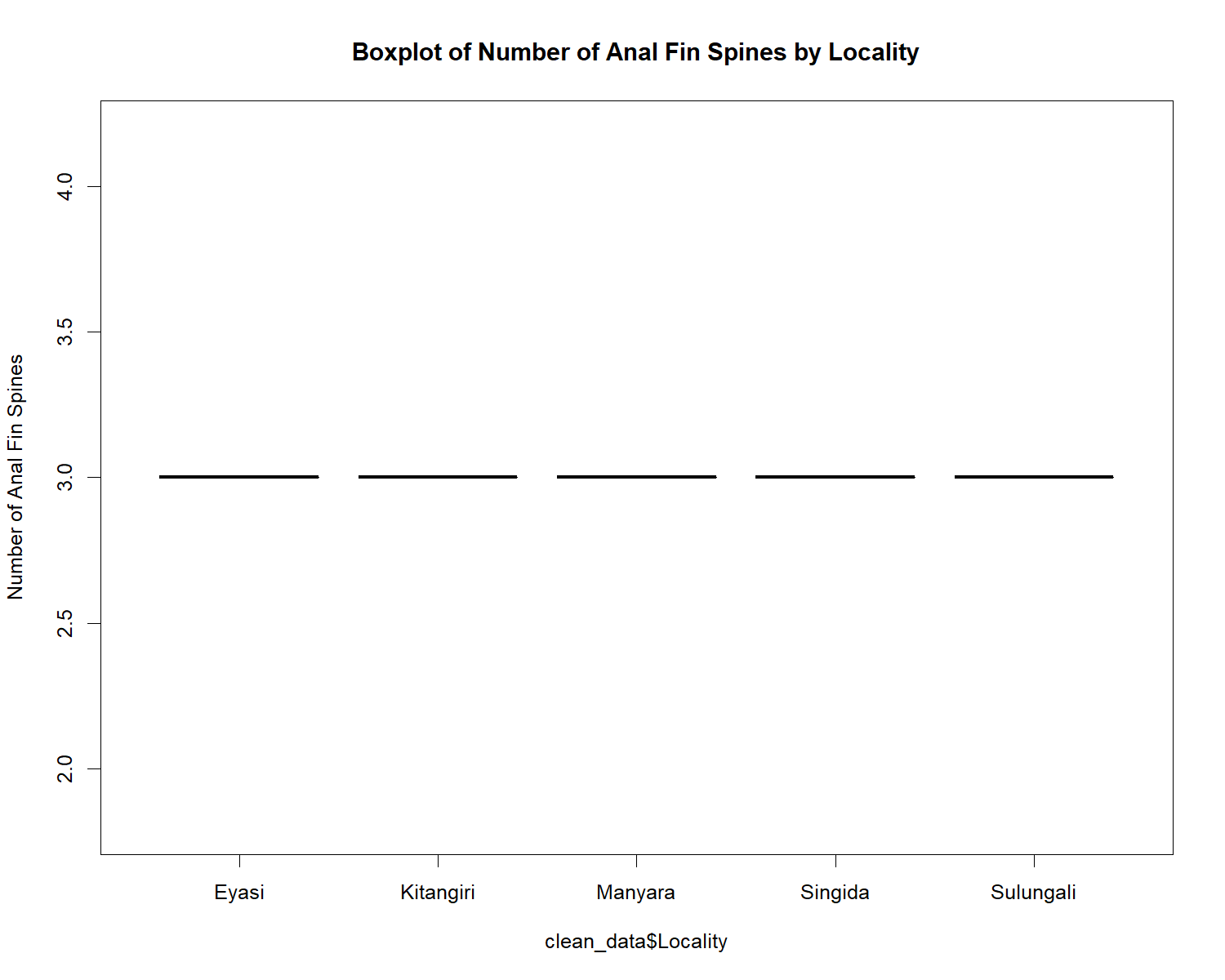

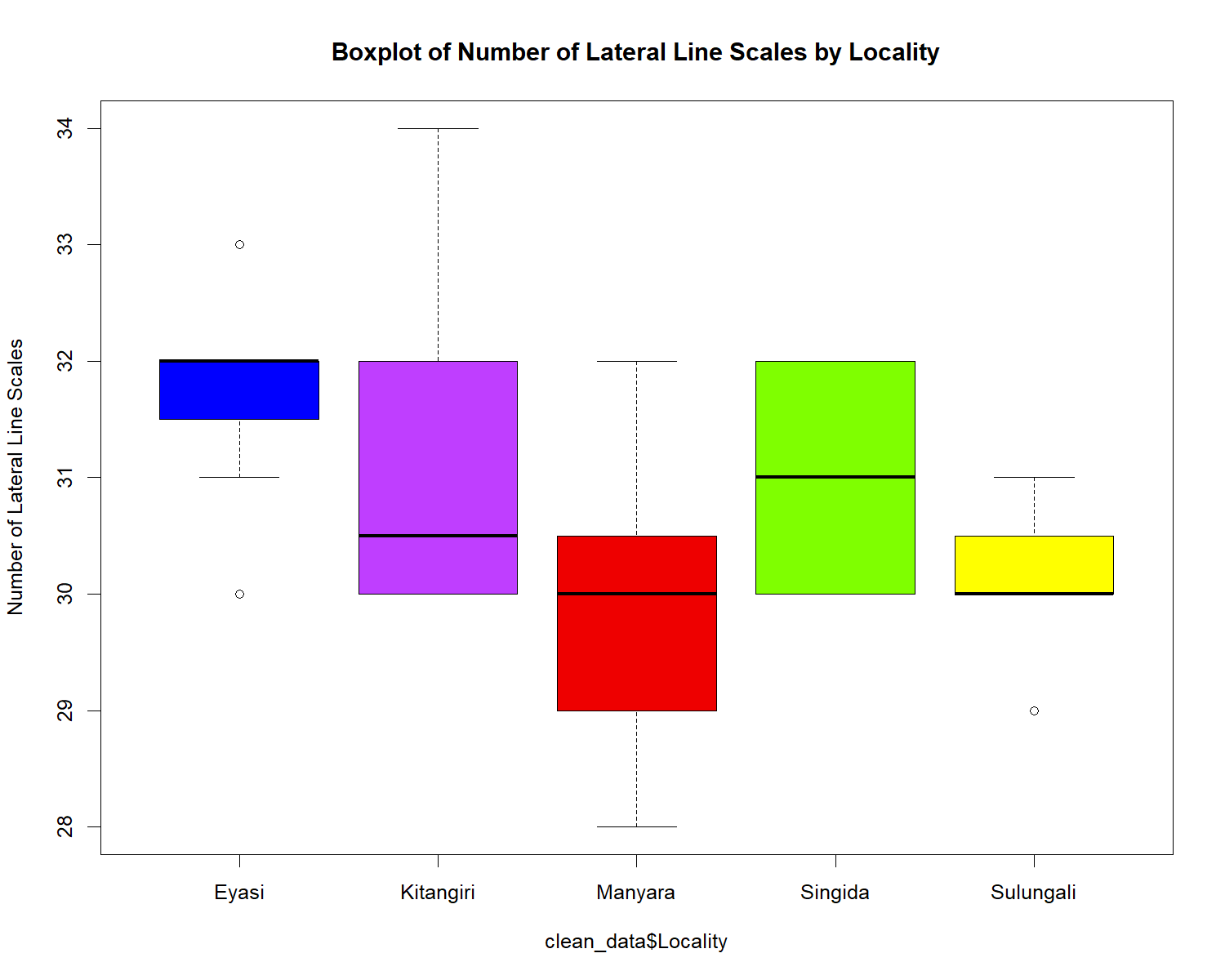


E F


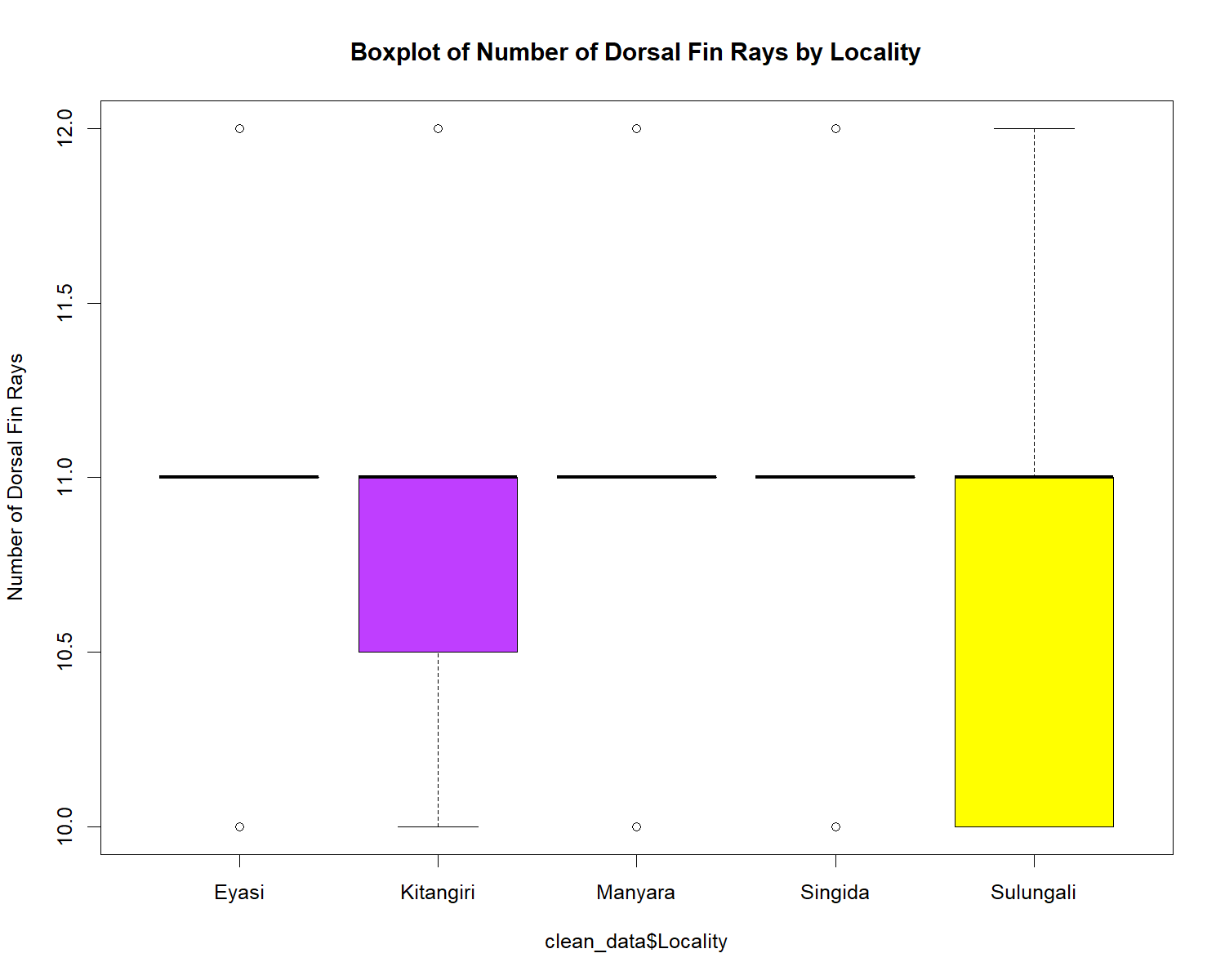

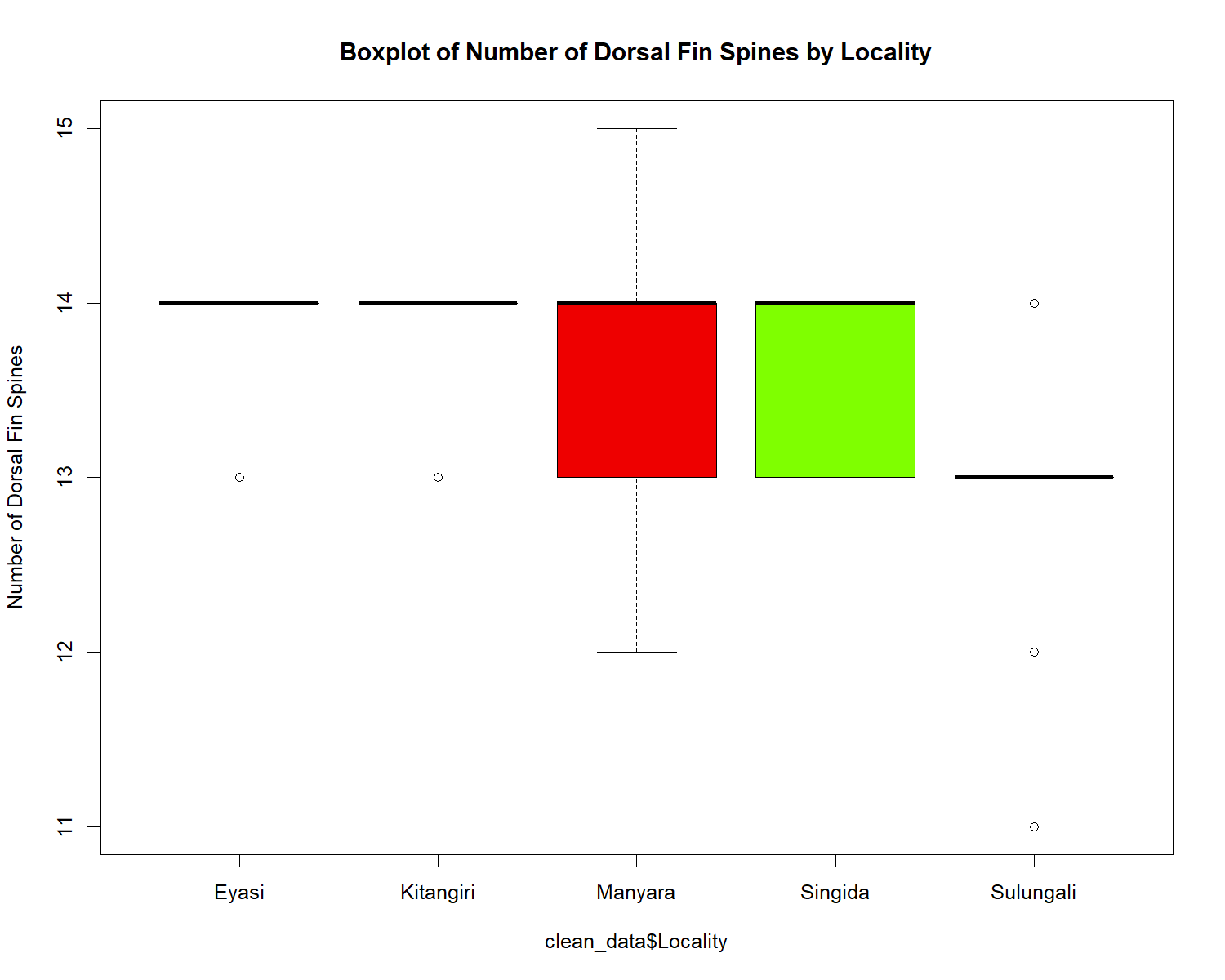


G H


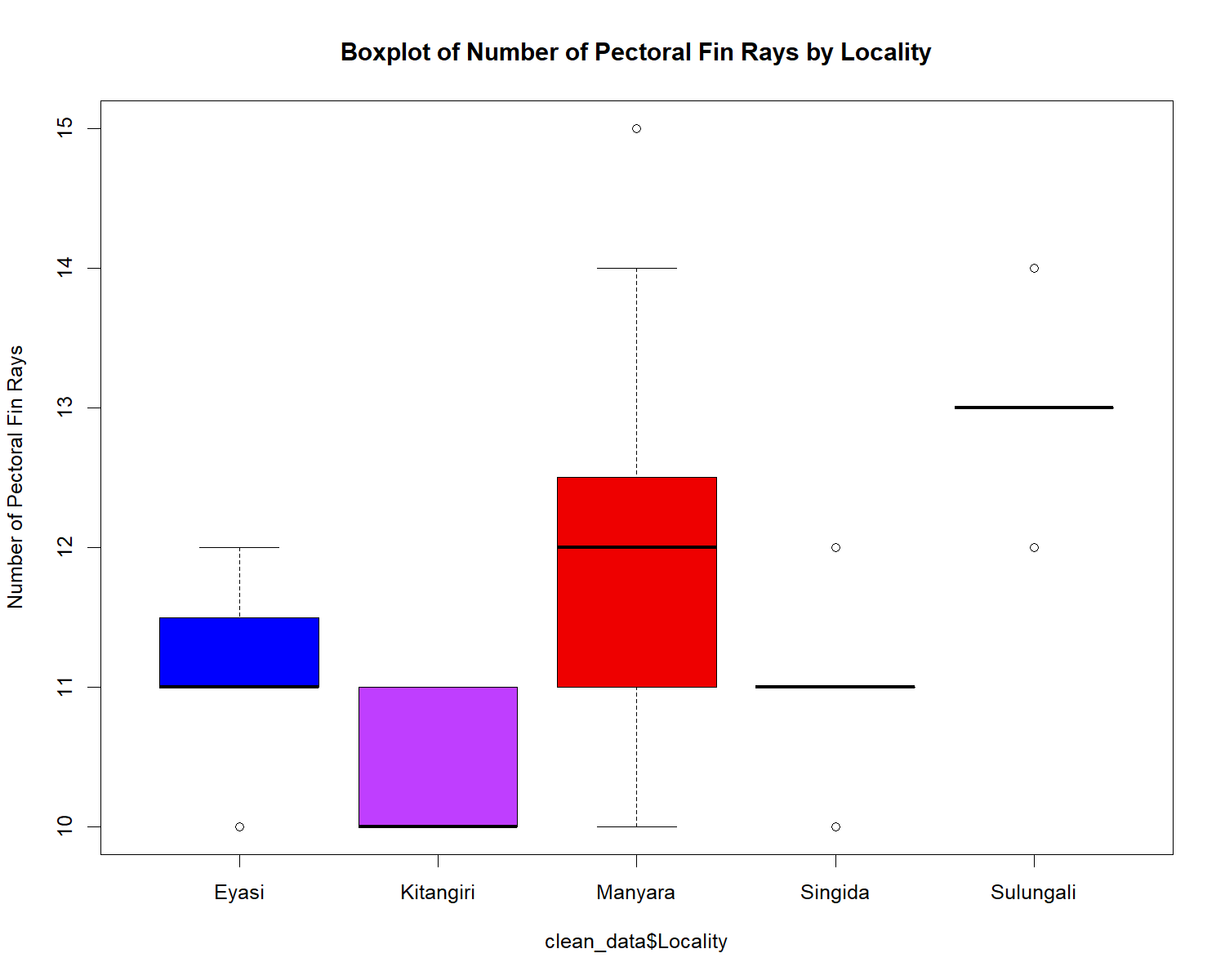

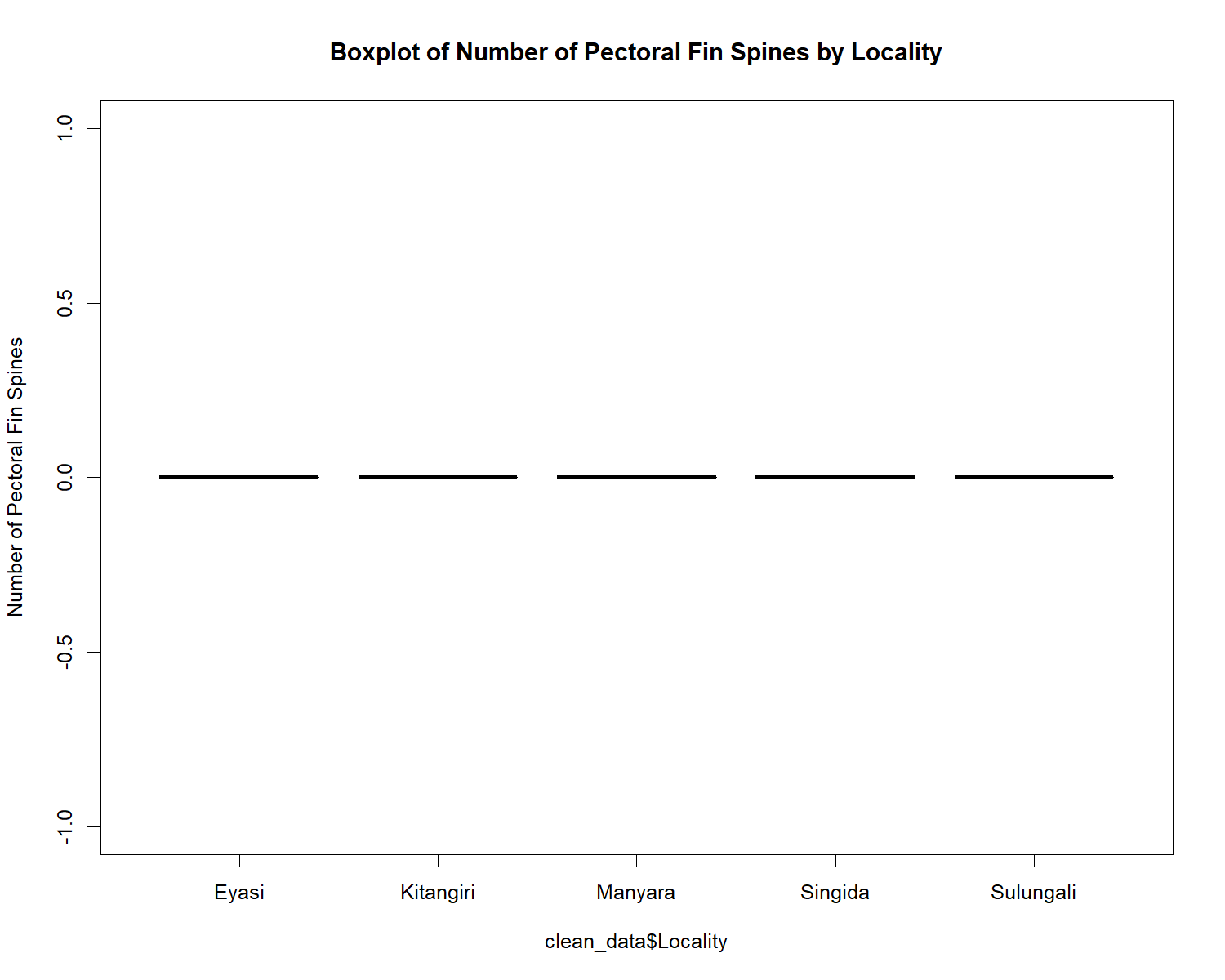


I J


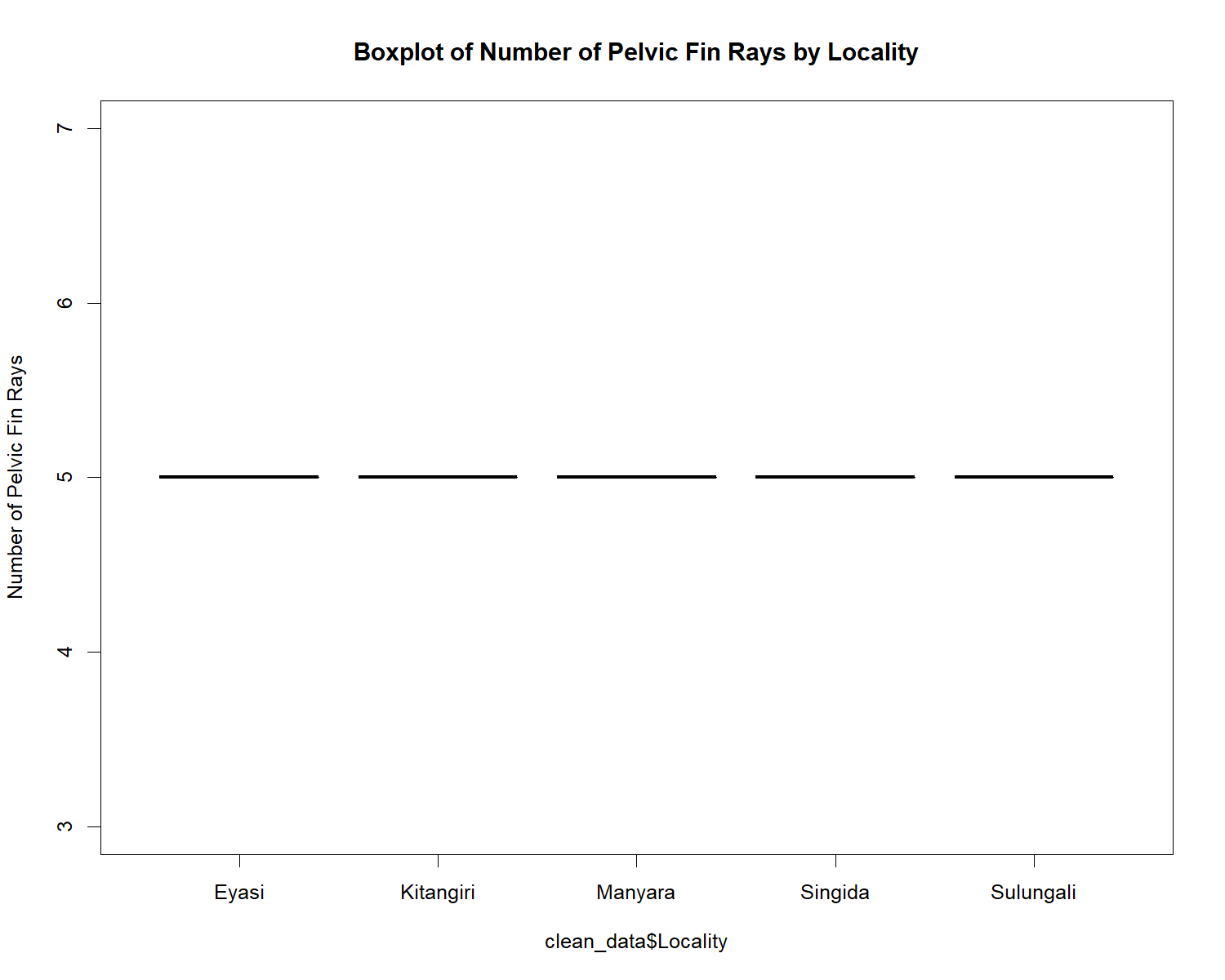

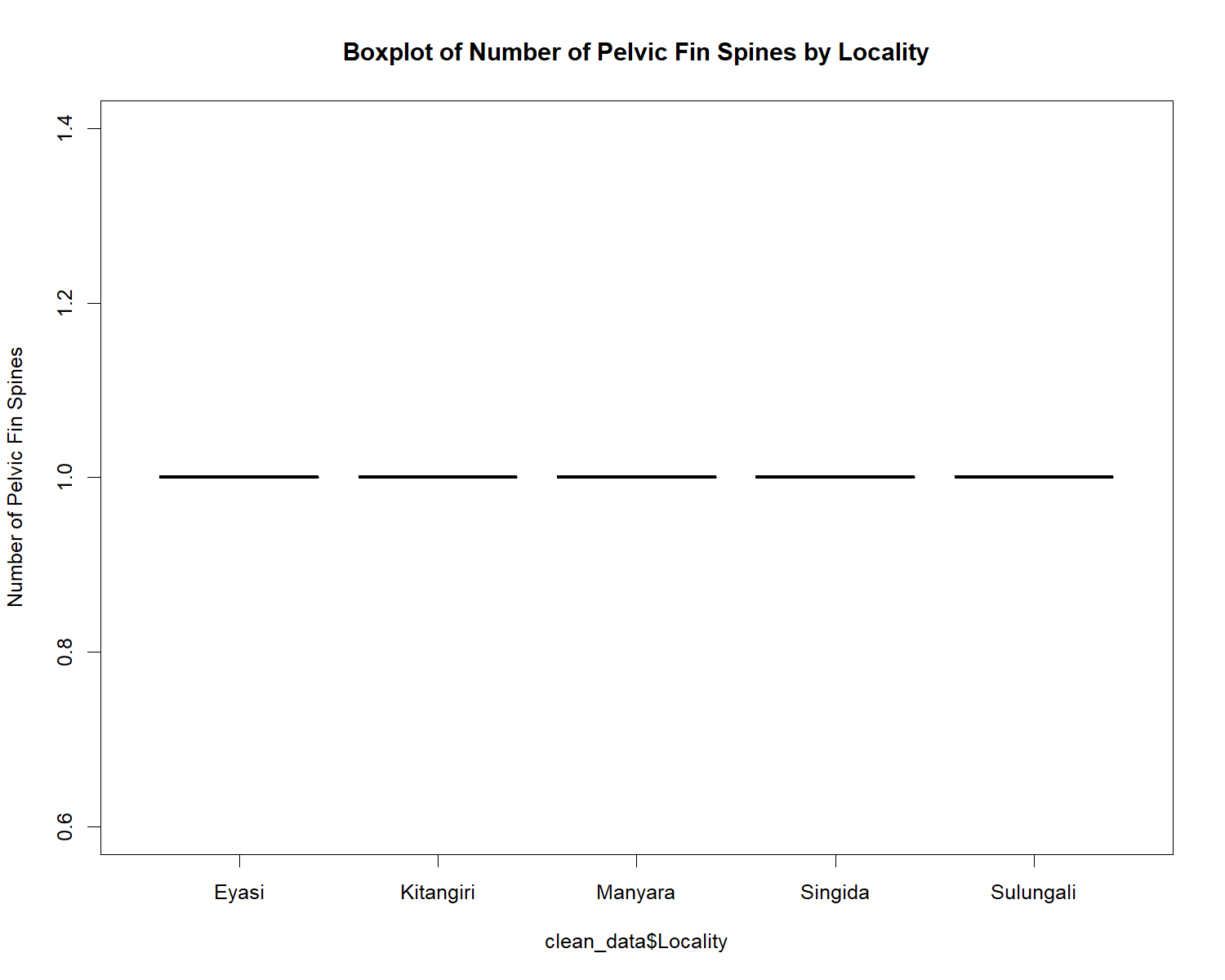


**Figure S7**. Ranges (upper and lower limits of boxes) and median values (line within boxes) of the measures and meristic counts that showed variation within the complete *Oreochromis amphimelas* dataset. A) Standard length, B) Head length, C) Anal fin spines, D) Lateral line scales, E) Dorsal fin rays, F) Dorsal fin spines, G) Pectoral fin rays, H) Pectoral fin spines, I) Pelvic fin rays, J) Pelvic fin spines.
